# Hospital-Level Care at Home for Adults Living in Rural Settings: A Randomized Clinical Trial

**DOI:** 10.1001/jamanetworkopen.2025.45712

**Published:** 2025-12-01

**Authors:** David M. Levine, Meghna P. Desai, Sarah M. Findeisen, Stephanie C. Blitzer, Ryan C. L. Brewster, Michelle N. Grinman, Steven C. Amrhein, Mitchell Wicker, Scott M. Harrison, Patricia C. Dykes, Mary Frances Barthel, Stuart R. Lipsitz

**Affiliations:** 1 Division of General Internal Medicine and Primary Care, Brigham and Women’s Hospital, Boston, Massachusetts; 2 Harvard Medical School, Boston, Massachusetts; 3 Ariadne Labs, Boston, Massachusetts; 4 Department of Neonatology, Beth Israel Deaconess Medical Center, Boston, Massachusetts; 5 Alberta Health Services, Calgary, Alberta, Canada; 6 Alberta Health Services, Edmonton, Alberta, Canada; 7 Department of Medicine, Cumming School of Medicine, University of Calgary, Calgary, Alberta, Canada; 8 Hazard Appalachian Regional Healthcare Regional Medical Center, Hazard, Kentucky; 9 The Center for Patient Safety Research and Practice, Brigham and Women’s Hospital, Boston, Massachusetts; 10 Blessing Health System, Quincy, Illinois

## Abstract

**Importance:**

Home hospital provides hospital-level care at home for patients with acute illness who would traditionally be cared for in a brick-and-mortar (BAM) hospital. While most home hospital programs have been implemented in urban areas, its feasibility in rural areas, where access to care is a major challenge, is unknown.

**Objective:**

To compare home hospital care with BAM hospital care for patients residing in rural areas.

**Design, Setting, and Participants:**

This randomized clinical trial took place from 2022 to 2023 with a 30-day follow-up in 3 rural areas in the US and Canada. Participants were adults recruited in the emergency department who required hospital-level care for select acute conditions (infections, heart failure, chronic obstructive pulmonary disease or asthma, and other diagnoses).

**Interventions:**

Patients in the home hospital group received acute care at home, including in-home nurse and/or paramedic visits, remote physician care, intravenous medications, remote monitoring, video communication, and point-of-care testing. Patients in the BAM group received services at a rural BAM hospital.

**Main Outcomes and Measures:**

The primary outcome was the relative change in the acute care episode’s direct cost. Secondary outcomes were 30-day readmission, days at home within 30 days of discharge, and physical activity. Exploratory outcomes included the Picker Experience Score and Net Promoter Score.

**Results:**

A total of 161 patients (79 home; 82 BAM) with mean (SD) age of 64.4 (17.2) years (home) and 64.9 (14.1) years (control) were included. Most were female (home, 52 [65.8%]; BAM, 50 [61.0%]). The adjusted mean cost of the acute episode was not significantly different (home vs BAM, 14% greater; 95% CI, −6% to 39%; *P* = .19). There were no significant differences in 30-day readmission (home vs BAM: 8 [10.1%] vs 14 [17.1%]) or mean (SD) days at home within 30 days of discharge (home vs BAM: 28.6 [3.4] vs 28.4 [3.4] days). Patients in the home hospital group were less sedentary, according to accelerometer measurements, than those in the BAM group (mean [SD], 78.0% [10.4%] vs 86.0% [7.2%] of the day sedentary; mean difference, −8.0%; 95% CI, −12.8% to −3.3%; *P* < .001) and had more mean (SD) steps daily (834.1 [1219.6] vs 120.4 [206.0] steps; mean difference, 713.7 steps; 95% CI, 290.2 to 1137.2 steps; *P* < .001). Total mean (SD) length of stay (ie, BAM and home hospital days for intervention patients and BAM days for control patients) was not significantly different (home vs BAM: 6.7 [5.0] days vs 5.4 [4.4] days), although patients receiving care at home transferred late in their course (mean [SD] day of transfer, 4.2 [4.3] of 6.7 days). Patients in the home hospital group reported better experiences than those in the BAM hospital group: the mean (SD) Picker experience score was 13.4 (2.6) vs 11.0 (3.8) (mean difference, 2.4; 95% CI, 1.0 to 3.8; *P* < .001), and the mean (SD) net-promoter score was 88.4 (32.3) vs 45.5 (69.9) (mean difference, 43.0; 95% CI, 17.5 to 68.5; *P* < .001). Safety events occurred in 11 (14.1%) home patients vs 10 (12.4%) BAM patients (mean difference, 1.8%; 95% CI, −8.1% to 11.6%; *P* = .74).

**Conclusions and Relevance:**

In this randomized clinical trial of home hospital care in rural settings, cost and readmission were unchanged while patient activity and experience improved. Late transfer home likely attenuated the intervention’s effect.

**Trial Registration:**

ClinicalTrials.gov Identifier: NCT05256303

## Key Points

**Question:**

For patients living in rural areas, how do cost, 30-day readmission rates, physical activity levels, and patient experience differ between those receiving acute care at home (home hospital) and those receiving care in a traditional brick-and-mortar hospital?

**Findings:**

In this 3-site randomized clinical trial of 161 adult patients living in rural areas, there were no significant adjusted mean differences in cost or 30-day readmission rates, but patients who received home hospital were more physically active and strongly preferred it over brick-and-mortar hospital care. As implemented, patients were transferred home late in their course (mean day of transfer, 4.2 of 6.7 days), likely attenuating the effect of the intervention on cost and readmission.

**Meaning:**

These findings suggest that home hospital care in rural settings is a feasible care model for acutely ill adults.

## Teaser

This randomized clinical trial investigates home hospital care as a feasible alternative to brick-and-mortar hospital care for acutely ill adults in rural settings.

## Introduction

About 14% of US residents and 18% of Canadians live in rural areas, totaling nearly 52 million people.^1,2^ For rural US residents, 23% say access to health care is a major problem and a similar percentage report an average 34-minute drive to their nearest brick-and-mortar (BAM) hospital.^3^ Poor access is likely to intensify given that rural hospitals are closing in record number in the US: 150 since 2010.^4^ Those who receive care in a hospital may encounter an environment that is potentially unsafe, of poor quality or experience, and expensive,^5,6^ particularly in rural hospitals.^7,8,9^

Acute hospital care at home, or home hospital, is the substitutive provision of home-based acute care services usually associated with a BAM hospital.^10,11^ Typically, patient care includes twice daily nurse or mobile integrated health paramedic visits; once daily physician visits; biometric monitoring; 24 hours a day, 7 days a week in-home response; and portable diagnostics. Prior research demonstrates that compared with BAM care, patients with acute illness residing in urban areas and cared for at home have improved experiences and levels of physical activity, with lower mortality, cost, rates of readmission, and discharge to skilled nursing facilities.^12,13,14,15,16,17,18,19,20^ National data show home hospital enables equitable care delivery among patients with disability, dual Medicare and Medicaid eligibility, and belonging to historically marginalized racial or ethnic populations .^21,22,23^

The majority of home hospital care has been delivered in urban centers, where access to supplies, people, and infrastructure is more facile. Given the substantial effort needed to coordinate hospital care at home, it is unclear whether rural settings can effectively support this model and achieve similar improved outcomes compared with BAM hospital care. We sought to demonstrate the efficacy of rural home hospital (RHH) compared with usual care.

## Methods

### Design Overview

We performed a parallel-design, randomized clinical trial with participants randomly allocated to RHH (intervention) vs BAM hospital (control). It was not possible to blind patients, study staff, or clinical care team members to allocation status, although we used scripting for all telephone surveys; the final assessor of outcomes (D.M.L.) was blinded. We enrolled participants at 3 sites in the US and Canada between February 23, 2022, and December 17, 2023; follow-up ended January 17, 2024, 30 days after the last patient was discharged. The trial protocol (see Supplement 1) was approved by the Mass General Brigham institutional review board and registered at ClinicalTrials.gov. US sites ceded to the Mass General Brigham institutional review board; the Canadian site was approved by the University of Calgary Conjoint Human Research Ethics Board. All participants provided written informed consent prior to enrollment. This trial follows the Consolidated Standards of Reporting Trials (CONSORT) reporting guidelines.

### Settings and Participants

Adult participants were recruited in the emergency department (ED) or medical ward at Blessing Hospital (Quincy, Illinois), Hazard Appalachian Regional Healthcare Regional Medical Center (Hazard, Kentucky), and Wetaskiwin Hospital and Care Centre (Wetaskiwin, Alberta, Canada). We recognize the definition of rural varies. We used prespecified criteria from the Federal Office of Rural Health Policy (US) and Alberta Health Services; all study sites met these criteria (eTable 1 in Supplement 2). Supplement 2 contains detailed criteria and site characteristics (eTables 1-3 in Supplement 2).

Patients were eligible to enroll only if they required inpatient admission using criteria already in use at the hospital. For US sites, existing hospital leveling criteria were used, and all patients were required to meet criteria for inpatient or acute care. Patients requiring only observation-level care were ineligible. At the Canadian site, local admission practices were maintained (leveling criteria were not relevant). Patients at all sites had to meet diagnosis-based criteria previously used to prognosticate against use of intensive care for conditions such as non–COVID-19 pneumonia or exacerbation of heart failure and live within a program’s specific catchment area (eTable 4 and eTable 5 in Supplement 2).^20^ Patients were ineligible if they required critical care, routine administration of controlled substances, an invasive procedure, more than 1-person assist to reach a bedside commode, or advanced imaging, among other criteria (eTable 4 in Supplement 2). US sites generally recruited patients with Medicare insurance due to the Acute Hospital Care at Home Waiver,^24^ although there was some operational variation. Canadian patients were required to have an Alberta Health Care Insurance Program number. Patients could live alone. We approached all eligible patients.

### Randomization

Eligible participants who provided informed consent were randomized by research study staff to RHH (home group) vs BAM (control group). Patients in the ED were randomized only after their admission was confirmed; patients receiving care on a BAM general ward could be randomized at any point during their hospital stay. Randomization was stratified by infection, heart failure, chronic obstructive pulmonary disease and/or asthma, and other diagnosis, with randomly selected block sizes between 4 and 6 with allocation concealment. Randomization was performed using the Research Electronic Data Capture (REDCap) randomization module.

### Intervention

With the important exception of physician care, we generally maintained our previously reported home hospital intervention^20^ with some site variation (eTable 5 in Supplement 2). Hospitalist attending physicians performed an initial in-hospital admission visit followed by a daily remote visit facilitated by in-home clinicians. At US sites, nurses conducted twice daily in-home visits. At the Canadian site, mobile integrated health paramedics performed once daily in-home visits, with a second visit either by video or in-home as needed by the patient (eTable 5 in Supplement 2). Training for physicians, nurses, and medics involved a 2-day remote didactic on best practices in home hospital medicine and the research protocol. Physicians were not given specific protocols for patient treatment. We cataloged various process measures specific to the intervention (eTable 6 in Supplement 2).

### Outcomes and Follow-Up

For both groups, study staff surveyed patients on admission, at discharge, and 30 days after discharge. On admission, patients reported their sociodemographic characteristics and completed assessments of frailty,^25^ cognitive impairment,^26^ depression,^27^ emotional support,^28^ health literacy,^29^ quality of life,^30^ and functional status.^31^ Participants self-reported their race and ethnicity. Race and ethnicity categories were investigator derived and included Asian, Black, Latino/Latina, multiracial or biracial, White, and other. Other races and ethnicities included Middle Eastern and Native people. Race and ethnicity were assessed in the study to help assess for generalizability. Study staff collected information from clinical staff and the electronic health record (EHR) for all other variables.

Our prespecified primary outcome was the total direct cost of the acute care episode, hereafter referred to as cost. Direct cost is cost attributable directly to the patient’s care; the cost of items that were paid for by the institution but are not necessarily directly applicable to a specific patient (for example, executive salaries) were not included in either group. Using the same method as in prior work,^20^ we calculated cost by summing the cost of nonphysician labor, supplies, monitoring equipment, medications, laboratory orders, radiology studies, and transport related to each patient’s care during the hospitalization (eTable 7 and eAppendix 1 in Supplement 2). We chose to focus on cost due to its importance on policymaking and that lower or similar cost would both be favorable toward the model’s viability.

Our prespecified secondary outcomes were total direct cost from discharge to 30 days postdischarge, unplanned readmission within 30 days of discharge, days at home (the number of days spent at home from the day of discharge to 30 days later), and percentage of day lying down (eAppendix 1 in Supplement 2). We also measured the Picker Patient Experience Questionnaire–15 (score, 0-15; higher indicating more positive experience),^32^ and global experience (scale, 0-10; higher indicating more positive experience) which we converted to a net promoter score.^33,34,35^ If patients were unable to be reached after discharge (26 total patients; control group: 14, home group: 12), we used EHR data alone and did not measure patient experience. We explored multiple additional quality, safety, and utilization metrics (eTable 8 in Supplement 2).

### Statistical Analysis

Patient characteristics are described using percentages for nominal categorical variables and means for ordinal categorical variables and continuous variables. To check for balance in these patient characteristics across treatment arms, Rao-Scott χ^2^ tests, accounting for site, were used for nominal categorical variables and Wilcoxon rank sum tests, accounting for site, were used for comparing ordinal or continuous variables.

The primary outcome was assessed using a generalized linear model assuming a γ distribution with a log link, given the skewed nature of cost, with treatment group and site as fixed effects. We present cost data as the percentage of change in mean cost from control (rather than absolute difference) because of the sensitive nature of these data. The exponential of the treatment effect of this γ regression model exactly equals the effect of interest (the percentage change from control). We a priori had planned to adjust for differences in patient characteristics, COVID-19 case burden, and degree of rurality, but these were not different after randomization.

We used this same multivariable regression approach for secondary outcomes, using logistic regression for binary outcomes (eg, readmission) and negative binomial regression for count outcomes, such as days at home. Given the multiple comparisons, we used the Benjamini-Hochberg method for multiple comparisons, with 0.2 as a prespecified false discovery rate. This adjustment led to no differences in our conclusions, and so we present the raw *P* values throughout. On the basis of data from our previous randomized clinical trial of urban home hospital care, we expected the mean cost of the intervention to be 40% lower than control (leading to a ratio of mean cost in treatment to control to be at most 0.6).^20^ We therefore required 132 patients total (66 in each group and 44 total per site) to detect a 40% relative decrease in mean direct cost in home vs control (power, 80%; α = .05, 2-tailed) using the Wald test for the treatment effect from a γ regression model (over-dispersion, 1.2) for costs discussed previously.

Due to implementation patterns that resulted in predominantly late transfers to the home of patients in the home group, we first performed a post hoc analysis that compared control patients with home patients who transferred home after less than 24 hours in BAM, after 1 to 2 days in BAM, and after 3 or more days in BAM. We demonstrated a dose-response for nearly all outcomes (ie, fewer days in the BAM led to better outcomes). For interpretability, we present here a post hoc analysis that compared control patients with home patients who transferred home in less than 3 days of BAM time. All analyses are intention to treat. We performed all analyses in R studio version 4.3.1 (R Project for Statistical Computing) and SAS version 9.4 (SAS Institute) from January to December 2024.

## Results

### Patient Characteristics

Of the 257 patients assessed for eligibility, 165 were enrolled and randomized (82 home, 83 control) (Figure). Of the 257 assessed for eligibility, 75 patients (31.3%) declined enrollment. Nearly all patients who enrolled received their allocated intervention (77 home; 80 control).

**Figure.**
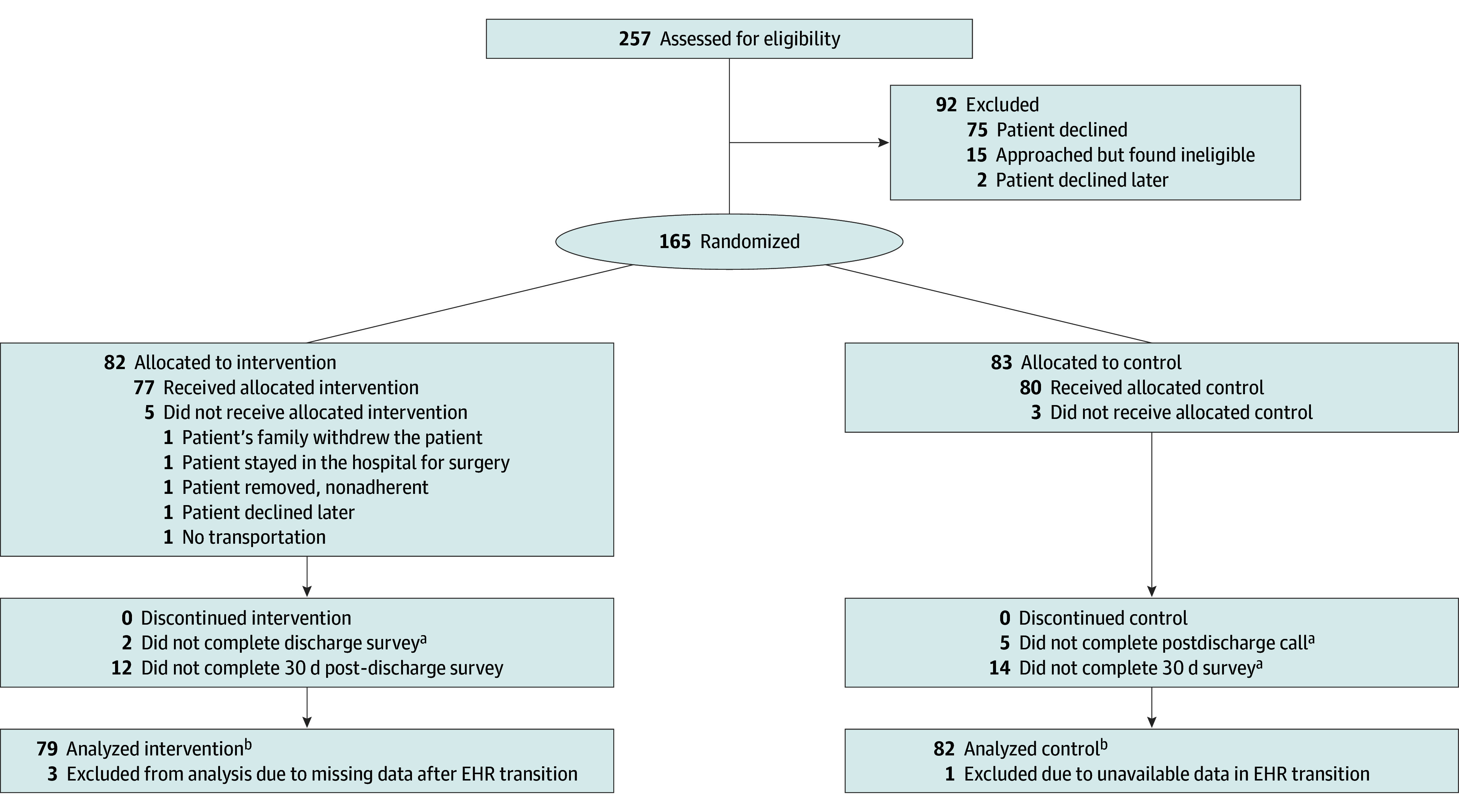
Participant Flow Diagram EHR indicates electronic health record. ^a^Not completing a postdischarge call incurred missing values for patient experience. ^b^Accrual by site was Appalachian Regional Medical Center, 77 patients (48%); Blessing Health System, 51 patients (32%); and Alberta Health Services, 33 patients (20%).

Patients were generally older (mean [SD], 64.4 [17.2] years home and 64.9 [14.1] years control), frail (mean [SD] PRISMA frailty score, 2.7 [1.6] home and 2.7 [1.6] control), chronically ill adults (mean [SD] comorbidity count, 3.9 [2.4] home and 4.0 [2.1] control), and were similar in both groups (Table 1). Most patients were female (52 [65.8%] home and 50 [61.0%] control), identified as White (77 [97.5%] home, 76 [92.7%] control), and spoke English (100% home and control).

**Table 1.** Baseline Patient Characteristics^a^

Characteristic	Participants, No. (%)	
	Home (n = 79)	Control (n = 82)
Age, mean (SD), y	64.35 (17.22)	64.94 (14.11)
Sex		
Female	52 (65.82)	50 (60.98)
Male	27 (34.18)	32 (39.02)
Race and ethnicity		
Asian	1 (1.27)	0
Black	1 (1.27)	2 (2.44)
Latino and/or Latina	0	0
Multiracial or biracial	0	0
White	77 (97.47)	76 (92.68)
Other^b^	0	4 (4.88)
Partner status		
Single	12 (15.19)	11 (13.41)
Married or life-partner	38 (48.10)	43 (52.44)
Divorced	13 (16.46)	11 (13.41)
Widowed	15 (18.99)	15 (18.29)
Other	1 (1.27)	2 (2.44)
Lives alone	20 (25.64)	22 (27.16)
Had home health aide	3 (3.85)	2 (2.47)
Primary language English	79 (100.00)	82 (100.00)
Health insurance^c^		
Private	3 (3.80)	6 (7.32)
Medicare	48 (60.76)	48 (58.54)
Medicaid	17 (21.52)	15 (18.29)
Medicare and Medicaid	11 (13.92)	12 (14.63)
None	0	1 (1.22)
Education		
Less than high school	16 (20.51)	17 (20.73)
High school	37 (47.44)	37 (45.12)
Completed some college	9 (11.54)	18 (21.95)
Associate’s degree	9 (11.54)	3 (3.66)
Bachelor’s degree (4-y)	6 (7.69)	5 (6.10)
Master’s degree or higher	1 (1.28)	2 (2.44)
Employment		
Employed	16 (20.25)	14 (17.07)
Unemployed	17 (21.52)	11 (13.41)
Retired	41 (51.90)	45 (54.88)
Other	5 (6.33)	12 (14.63)
Smoking status		
Yes	28 (35.44)	28 (34.15)
No	46 (58.23)	53 (64.63)
Do not know	5 (6.33)	1 (1.22)
Diagnosis		
Pneumonia	16 (20.25)	15 (18.29)
Chronic obstructive pulmonary disease	15 (18.99)	18 (21.95)
Other infection	14 (17.7)	17 (20.7)
Heart failure	11 (13.92)	12 (14.63)
Cellulitis	10 (12.66)	11 (13.41)
Complicated urinary tract infection and/or pyelonephritis	8 (10.13)	8 (9.76)
Asthma	4 (5.06)	0
Other^d^	1 (1.27)	1 (1.22)
Comorbid condition count at admission, mean (SD)^e^	3.92 (2.39)	3.95 (2.08)
PRISMA frailty score (SD) at admission, mean (SD)^f^	2.68 (1.63)	2.68 (1.61)
8-Item Interview to Differentiate Aging and Dementia score at admission, mean (SD)^g^	1.38 (2.12)	1.39 (2.21)
PHQ-2 score admission, mean (SD)^h^	1.43 (1.59)	1.30 (1.59)
PROMIS emotional support score at admission, mean (SD)^i^	58.54 (7.71)	58.66 (7.01)
Brief Health Literacy Screening Tool score at admission, mean (SD)^j^	15.78 (3.80)	16.05 (4.23)
EuroQol VAS score at admission, mean (SD)^k^	60.27 (21.58)	56.63 (20.01)
ADLs on at admission, mean (SD)^l^	5.53 (1.25)	5.60 (1.25)
IADLs on at admission, mean (SD)^m^	6.61 (2.24)	6.64 (2.06)
Full code status	72 (93.51)	77 (95.06)
Outpatient medications, mean (SD), No.	6.90 (5.28)	7.25 (6.45)
Readmission risk score on discharge, mean (SD)^n^	3.77 (2.01)	3.39 (1.92)
Admitted to hospital in past 6 mo	33 (42.31)	33 (40.24)
Visited ED in past 6 mo	50 (63.29)	50 (60.98)

Abbreviations: ADL, activity of daily living; ED, emergency department; IADL, instrumental ADL; PHQ-2, Patient Health Questionnaire 2; PRISMA, Program of Research to Integrate the Services for the Maintenance of Autonomy; PROMIS, Patient-Reported Outcomes Measurement Information System; VAS, visual analogue scale.

^a^ Percentages may not sum to 100 due to rounding. Missing data for living alone, having a home health aide, education, comorbidity count, PRISMA frailty score, 8-Item Interview to Differentiate Aging and Dementia score, PHQ-2 score, PROMIS score, health literacy score, EuroQol VAS score, ADLs, IADLs, and past 6-month hospital admissions are described in detail in eAppendix 2 in Supplement 2.

^b^ Other includes those who identified as Native people and Middle Eastern.

^c^ Medicare counts include Canadian provincial insurance.

^d^ Includes a viral illness and unknown diagnosis.

^e^ A count of the patient’s chronic comorbid conditions, out of the 20 conditions considered chronic by the Health and Human Services Office of the Assistant Secretary of Health.^36^

^f^ Range, 0 to 7, where scores greater than 2 indicate frailty.

^g^ Range, 0 to 8, where scores greater than 1 indicate cognitive impairment.

^h^ Range, 0 to 6, where scores greater than 2 indicate depression.

^i^ Range, 25.7 to 62, where scores greater than 50.8 indicate better-than-average emotional support.

^j^ Range, 4 to 20, where scores of 4 to 12 indicate limited health literacy, scores of 13 to 16 indicate marginal health literacy, and scores of 17 to 20 indicate adequate health literacy.

^k^ Range, 0 to 100, where higher scores indicate better health.

^l^ Range, 0 to 6, where higher scores indicate more independence.

^m^ Range, 0 to 8, where higher scores indicate more independence.

^n^ Range, 0 to 13, where scores of 0 to 4 indicate low risk of 30-day readmission, scores of 5 to 6 indicate intermediate risk of 30-day readmission, and scores greater than 7 indicate a high risk of 30-day readmission.

Forty-eight patients (29.8%) were admitted from the ED; 112 patients (69.6%) were transferred from the ward. One patient’s admission source was unknown. Diagnoses were similar in each group (Table 1). Most common was non–COVID-19 pneumonia, followed by exacerbation of chronic obstructive pulmonary disease, other infection, and then exacerbation of heart failure.

### Cost and Utilization

There were no statistically significant differences for cost between the groups after adjustment (Table 2). Mean unadjusted cost of the acute care episode was 6.8% higher (95% CI, −12.7% to 30.6%; *P* = .52) for home patients than control patients. Adjusted mean cost was 14.2% higher (95% CI, −6.3% to 39.2%; *P* = .19). In the 30 days after the acute care episode, the adjusted mean cost was 4.7% lower (95% CI, −59.4% to 123.7%; *P* = .91) lower for home patients.

**Table 2.** Relative Cost of Home Hospital Care to Brick-and-Mortar Hospital Care

Cost	Change, % (95% CI)^a^	*P* value
Acute care episode		
Unadjusted cost	6.81 (−12.67 to 30.62)	.52
Adjusted mean cost^b^	14.18 (−6.34 to 39.19)	.19
30 d After acute care episode		
Unadjusted cost	−21.26 (−64.34 to 73.84)	.55
Adjusted mean cost^b^	−4.74 (−59.44 to 123.71)	.91
Acute care episode and 30 d after acute care episode		
Unadjusted cost	−2.78 (−28.06 to 31.38)	.85
Adjusted mean cost^b^	4.27 (−18.44 to 33.3)	.74

^a^ Calculated as (ratio of mean home to mean hospital – 1) × 100. If percentage of change is negative, home group costs less; if percentage of change is positive, control group costs less.

^b^ Adjusted for site as a fixed effect.

Mean (SD) total length of stay was 6.7 (5.0) days in the home group vs 5.4 (4.4) days in the control group (Table 3). Home patients transferred to home late in their acute care course, spending most of their acute care days in the BAM hospital (mean [SD], 4.2 [4.3] days), not at home (mean [SD], 2.4 [1.8] days). Patients had similar utilization, except home patients had fewer mean (SD) blood tests than control patients (6.0 [3.2] vs 9.2 [4.8]) per day. While 30-day utilization was not statistically significantly different, home patients had substantially fewer readmissions (8 [10.1%] vs 14 [17.1%]) and no difference in ED visits (16 [20.3%] vs 14 [17.1%]).

**Table 3.** Patient Health Care Use^a^

Measure	Participants, No. (%)		Difference, mean (95% CI), percentage points	*P* value
	Home (n = 79)	Control (n = 82)		
During acute care episode^b^				
Total LOS, mean (SD), d	6.67 (5.02)	5.38 (4.36)	1.29 (−0.16 to 2.74)	.08
LOS in brick-and-mortar, mean (SD), d	4.23 (4.31)	NA	NA	NA
LOS at home, mean (SD), d	2.44 (1.75)	NA	NA	NA
Intravenous medication^c^	78 (98.73)	77 (95.06)	3.67 (−2.17 to 9.51)	.22
Imaging^d^	56 (70.89)	61 (74.39)	−3.50 (−16.86 to 9.86)	.62
Oxygen requirement	44 (55.7)	48 (58.54)	−2.84 (−17.64 to 11.96)	.72
Nebulizer requirement^c^	35 (44.3)	47 (58.02)	−13.72 (−29.6 to 2.16)	.09
Laboratory orders per day, mean (SD)	6.02 (3.16)	9.16 (4.84)	−3.14 (−5 to −1.28)	<.001
Consultant sessions	35 (44.3)	49 (59.76)	−15.46 (−3.95 to 0.03)	.05
Physical or occupational therapy	19 (24.05)	20 (24.39)	−0.34 (−12.43 to 11.75)	.96
Disposition^c^				
Routine	66 (83.54)	66 (81.48)	2.06 (−4.46 to 8.58)	<.001
Home health	12 (15.19)	8 (9.88)	5.31 (−4.43 to 15.05)	
Home hospice	1 (1.27)	2 (2.47)	−1.20 (−5.79 to 3.39)	
Other^e^	NA	5 (6.17)	−6.17 (−11.72 to −0.62)	
30 d After acute care episode				
Days at home, mean (SD)	28.63 (3.44)	28.43 (3.43)	0.20 (−0.77 to 1.17)	.70
Unplanned readmission	8 (10.13)	14 (17.07)	−6.94 (−17.75 to 3.87)	.21
Unplanned readmission, mean (SD), No.	0.11 (0.36)	0.2 (0.46)	0.16 (−0.09 to 0.41)	.21
ED visit or ED observation	16 (20.25)	14 (17.07)	3.18 (−8.63 to 14.99)	.61
ED visit or ED observation, mean (SD), No.	0.29 (0.75)	0.22 (0.54)	−0.25 (−0.94 to 0.44)	.49
SNF utilization days, mean (SD)^f^	NA	NA	NA	NA
Home health utilization days, mean (SD)^g^	0.82 (2.85)	0.61 (2)	0.21 (−0.55 to 0.97)	.60
Follow-up appointment within 14 d of discharge^h^	38 (59.38)	36 (52.17)	7.21 (−9.67 to 24.09)	.41

Abbreviations: ED, emergency department; LOS, length of stay; NA, not applicable; SNF, skilled nursing facility.

^a^ Percentages may not sum to 100 due to rounding, See eTable 8 in Supplement 2 for comparison of home vs hospital in the intervention group.

^b^ For home patients, includes any time they spent in the brick-and-mortar hospital.

^c^ For 1 control patient, these data are missing.

^d^ Electrocardiograms are included in imaging.

^e^ Four patients left against medical advice and 1 patient was discharged to another inpatient facility.

^f^ For 9 control patients and 9 intervention patients, these data are missing.

^g^ For 4 control patients and 4 intervention patients, these data are missing.

^h^ For 13 control patients and 15 intervention patients, these data are missing.

Among home patients, cost and utilization were lower during their days at home compared with their days in the BAM (eTable 9 in Supplement 2). For example, adjusted mean (SD) cost per day was 49.4% less (95% CI, −60.6% to −35.0%) at home than in the BAM hospital. Patients required a mean (SD) of 1.8 (2.0) laboratory orders per day at home vs 7.6 (3.7) in the BAM.

### Safety, Quality, Physical Activity, Functional Status, and Experience

There were no appreciable differences in safety between the 2 groups (Table 4; eTable 8 in Supplement 2). At least 1 safety event occurred in 11 home patients (14.1%) vs 10 BAM patients (12.4%) (mean difference, 1.8%; 95% CI, −8.1% to 11.6%; *P* = .74). Patients in both groups saw frequent use of potentially inappropriate medications (home vs control, 41.8% vs 34.6%; mean difference, 7.2%; 95% CI, −7.7% to 22.1%). Use of urinary catheters was low in both groups. In the home group, 6 patients (7.6%) were transferred back to the BAM hospital during their admission to RHH. Reasons included patient nonadherence to the protocol, worsening symptoms, and fall.

**Table 4.** Quality, Safety, Physical Activity, Functional Status, and Experience^a^

Measure	Score, mean (SD)		Mean difference (95% CI)	*P* value
	Home (n = 79)	Control (n = 82)		
Quality and safety of care				
Any safety event, No. (%)^b^	11 (14.10)	10 (12.35)	1.75 (−8.07 to 11.57)	.74
Pain score^c^	2.12 (2.17)	2.88 (2.63)	−0.76 (−1.52 to 0)	.05
Potentially inappropriate medication use, No. (%)^d^	33 (41.77)	28 (34.57)	7.20 (−7.73 to 22.13)	.35
Urinary catheter use, No. (%)	5 (6.49)	3 (3.7)	2.79 (−4.02 to 9.6)	.43
Restraint use, No. (%)	1 (1.27)	NA	1.27 (−21.5 to 24.04)	.92
Activity each day				
Minutes of sedentary activity, %^e^	77.95 (10.39)	85.97 (7.17)	−8.02 (−12.78 to −3.26)	<.001
Minutes of LPA, %^e^	21.25 (9.64)	13.92 (7.07)	7.33 (2.98 to 11.68)	<.001
Minutes of MVPA, %^e^	0.80 (1.67)	0.10 (0.28)	0.70 (0.22 to 1.18)	.004
Daily steps	834.09 (1219.63)	120.39 (206.03)	713.70 (290.22 to 1137.18)	<.001
Functional status				
EQ-5D-5L^f^				
Admission	8.81 (3.36)	8.90 (3.16)	−0.09 (−1.02 to 0.84)	.86
Discharge	8.17 (3.39)	8.37 (3.15)	−0.20 (−1.24 to 0.84)	.72
30 d	8.77 (3.41)	9.02 (4)	−0.25 (−1.55 to 1.05)	.72
PHQ-2^g^				
Admission	1.43 (1.59)	1.30 (1.59)	0.13 (−0.34 to 0.6)	.60
Discharge	1.17 (1.37)	1.25 (1.27)	−0.08 (−0.47 to 0.31)	.70
30 d	1.27 (1.92)	0.95 (1.44)	0.32 (−0.29 to 0.93)	.31
ADLs^h^				
Admission	5.53 (1.25)	5.60 (1.25)	−0.07 (−0.42 to 0.28)	.71
Discharge, mean (SE)	5.42 (0.17)	5.48 (0.16)	−0.06 (−0.54 to 0.42)	.82
30 d, mean (SE)	5.30 (0.20)	5.25 (0.21)	0.05 (−0.43 to 0.53)	.85
IADLs^i^				
Admission	6.61 (2.24)	6.64 (2.06)	−0.03 (−0.64 to 0.58)	.93
Discharge	6.69 (2.16)	6.39 (2.26)	0.30 (−0.4 to 1)	.41
30 d	6.55 (2.42)	6.57 (2.06)	−0.02 (−0.59 to 0.55)	.95
Functional status change				
ADLs worse: admission to discharge, proportion (SE)^j^	0.15 (0.04)	0.10 (0.04)	0.05 (−7.01 to 7.11)	.99
ADLs worse: admission to 30 d after discharge, proportion (SE)^j^	0.18 (0.06)	0.21 (0.05)	−0.03 (−0.17 to 0.11)	.69
IADLs worse: admission to discharge, No. (%)	13 (20)	19 (26.39)	−6 (−18.95 to 6.95)	.37
IADLs worse: admission to 30 d after discharge, No. (%)	11 (21.57)	19 (31.67)	−8 (−21.28 to 5.28)	.24
Patient experience				
Picker patient experience questionnaire score^k^	13.41 (2.57)	11 (3.81)	2.41 (0.98 to 3.84)	<.001
Global satisfaction score^l^	9.44 (1.05)	8.34 (1.74)	1.10 (0.45 to 1.75)	<.001
Recommend care^m^	9.64 (0.8)	8.53 (1.82)	1.11 (0.45 to 1.77)	<.001
Net promoter score^n^	88.41 (32.25)	45.45 (69.86)	42.96 (17.47 to 68.45)	<.001

Abbreviations: ADL, activity of daily living; IADL, instrumental ADL; EQ-5D-5L, EuroQol-5 Dimensions-5 Levels; LPA, light physical activity; MVPA, moderate to vigorous activity; PHQ-2, Patient Health Questionnaire 2.

^a^ Missing data for any safety event, pain scores, urinary catheter use, minutes of activity each day, EQ-5D-5L functional status, PHQ-2 functional status, ADLs functional status, iADLs functional status, ADLs functional status change, IADLs functional status change, Picker patient experience questionnaire score, global satisfaction score, recommend care, and net promoter score, are described in detail in eAppendix 3 in Supplement 2.

^b^ See eTable 8 in Supplement 2 for detailed safety outcomes.

^c^ Range, 0 to 10 where higher scores indicate greater pain.

^d^ Using the updated Beers Criteria.^37^

^e^ Activity was categorized into sedentary, light, moderate, or vigorous with sedentary being the least physical activity and vigorous being the most physical activity.

^f^ Range, 5 to 25 where lower scores indicate less perceived health problems and higher scores indicate more perceived health problems.

^g^ Range, 0 to 6, where scores greater than 2 indicate depression.

^h^ Range, 0 to 6, where higher scores indicate higher patient independence.

^i^ Range, 0 to 8, where higher scores indicate higher function and independence.

^j^ These values were imputed and changed across datasets.

^k^ Range 0 to 15, where higher scores indicate more positive patient experience.

^l^ Range 0 to 10, where higher scores indicate higher global satisfaction.

^m^ Range 0 to 10, where higher scores indicate more likely to recommend.

^n^ Range, −100 to 100, where higher scores indicate more likely to recommend.

Home patients were significantly more physically active than control patients (Table 4). Home patients spent a smaller percentage of minutes per day sedentary than control patients (mean [SD], 78.0% [10.4%] vs 86.0% [7.2%] of the day sedentary; mean difference, −8.0%; 95% CI, −12.8% to −3.3%; *P* < .001) and walked more mean (SD) steps each day than control patients (834.1 [1219.6] vs 120.4 [206.0]; mean difference, 713.7 steps; 95% CI, 290.2 to 1137.2 steps; *P* < .001). We observed no differences between the groups in activities of daily living or instrumental activities of daily living.

Patient experience was significantly better in the home group (Table 4). Mean (SD) Picker experience score was 13.4 (2.6) vs 11.0 (3.8) (mean difference, 2.4; 95% CI, 1.0 to 3.8; *P* < .001). Mean (SD) global satisfaction score was 9.4 (1.1) vs 8.3 (1.7) (mean difference, 1.1; 95% CI, 0.5 to 1.8; *P* < .001), which rendered a mean (SD) net promoter score of 88.4 (32.3) vs 45.5 (69.9) (mean difference, 43.0; 95% CI, 17.5 to 68.5; *P* < .001).

### Post Hoc Analysis of Home Patients Transferred Home in Less Than 3 Days of BAM Care

Home patients who transferred home in less than 3 days of BAM care (n = 40) had similar sociodemographic characteristics as control patients (n = 82) (eTable 10 in Supplement 2). They had lower adjusted mean cost for the acute episode and 30-day episode combined than patients in the control group (27.1% lower; 95% CI, −44.8% to −3.6%; *P* = .03) (eTable 11 in Supplement 2). They also had less utilization: shorter mean (SD) length of stay (home vs control: 3.7 [1.7] vs 5.4 [4.4] day), fewer mean (SD) laboratory orders per day (home vs control: 6.1 [3.8] vs 9.2 [4.8]), and fewer consultations (home vs control 9 [22.5%] vs 49 [59.8%]) (eTable 12 in Supplement 2). They demonstrated similar improvements as the main analysis for physical activity and patient experience (eTable 13 in Supplement 2).

## Discussion

In this randomized clinical trial of adults with acute illness living in rural areas requiring hospital admission, we showed home hospital was a feasible care model to deliver hospital-level care in a patient’s home. RHH had no difference in cost or health care utilization, while significantly improving patient experience and physical activity. Post hoc hypothesis-generating analysis suggested that had patients transferred earlier from the BAM to home, we likely would have observed improvements in cost and utilization. Although our primary outcome was null, it is important to place these results in context for patients and policymakers: to our knowledge, there are few interventions that improve patient experience and physical activity without requiring a higher cost.

Compared with our prior randomized clinical trial^20^ that only enrolled patients directly from the emergency department, most patients in the home hospital group were transferred later in their hospital stay. This likely attenuated the effect of the home hospital intervention, particularly on cost and utilization, as longer BAM hospital stays meant longer time periods in a higher cost setting. Longer BAM time also potentially provided more opportunities for patients to develop hospital-acquired conditions, disability, infection, and overuse. One major factor was likely the high rate of specialist consultations, which were common even for routine cases. These consultations were likely associated with significant delays in enrolling patients in the home hospital program, as consultants were rarely available quickly in the rural setting, often only coming once or twice weekly to a hospital. Specialty teleconsultation or reduction in consultation could alleviate this. In a post hoc analysis where we analyzed only patients who transferred home in less than 3 days of BAM care, we found much more favorable outcomes. Patients who transferred earlier had similar sociodemographic and clinical characteristics to those who transferred later in their course. This suggests the timing of the transfer was more influenced by the process of care rather than differences in clinical complexity or acuity. Our power to detect statistically significant differences in this post hoc cohort was limited. This implementation pattern of late transfer serves as an important lesson for all home hospital programs: we likely cannot extrapolate results from randomized clinical trials that enrolled from the ED and expect a late transfer to be as efficacious for patients or hospitals. Future studies should report, measure, and potentially adjust for BAM days prior to transfer home.

Compared with urban studies, differences in this rural population may explain the different outcomes. First, it was a much younger sample, with a mean age of about 64 years compared with 70 to 80 years in most urban studies.^13,17^ This may explain why home hospital did not benefit some of our patients as much as would be expected. Some of the hospital-acquired conditions that home hospital has been shown to prevent, such as delirium, do not affect younger patients as often.^5^ Second, smoking rates were high, reflecting rates among the surrounding counties, and likely influenced high rates of chronic obstructive pulmonary disease exacerbations. It is possible that cigarette smoking attenuates the impact of home hospital perhaps because patients can more easily smoke when home.

We observed a larger acceptance of home hospital and better experience among rural patients. Rural patients were more accepting of the intervention, declining to enroll far less often (31%) than in our prior urban studies (63%).^16,20^ Quantitative patient experience had a similar pattern: no difference among urban patients (Picker experience score 14 in both group) vs significant improvements among rural patients (Picker Score 13 vs 11).^13^ Perhaps patients in rural areas experience much poorer access and quality care and therefore appreciated the benefits much more than urban patients.

Our rural model required some tradeoffs in clinical care that may have led to challenges with care delivery at home. First, following the admission encounter, all physician and advanced practice practitioner care was remote. Although this is now a practice in many urban home hospital programs, this likely limited the complexity and acuity of care that could be delivered. Second, some tools available in the urban setting were not available in the rural setting. For example, imaging and advanced vascular access placement required a round-trip to the BAM hospital. Third, there were multiple natural disasters involving catastrophic flooding and forest fires at 2 sites during enrollment, placing unique pressures on operations more often found in rural areas.

### Limitations

Our study has limitations. First, the 3 sites limit generalizability. Although geographically diverse and rural, nearly all patients were White and spoke English. Much of this was due to the location of the 3 sites; that is, few patients of other races and ethnicities lived in the surrounding counties (eTable 2 in Supplement 2). Patients in Alberta who lived on First Nation reserves were not eligible due to paramedic and physician payment and jurisdiction, preventing their representation. Second, our primary data sources were patients and the hospital EHR, not claims, which may have improved the capture of utilization, cost, and days at home in the post-acute 30-day period. However, both groups had this limitation. Third, our capture of some safety events was likely suboptimal. For example, we found zero incidence of delirium in either group, despite querying frontline clinicians and performing medical record review. This likely represents underreporting of a common adverse event in hospital medicine. Fourth, our power to detect small differences in cost was limited.

## Conclusions

In this randomized clinical trial of RHH, cost and readmission were unchanged, while patient activity and experience improved. The RHH model successfully delivered hospital-level care to patients living in rural areas without affecting cost while increasing patient physical activity and delivering a highly valued care experience. This study broadens the evidence base for home hospital to now include the 1 in 5 US residents living in rural areas. These data should inform the ongoing national and congressional dialogue for continued and one day permanent payment for hospital-level care at home.

## Notes

Supplement 1. Trial Protocol

Supplement 2. eTable 1. Definition of Rural eTable 2a. Geographic Details by Site eTable 2b. Additional Geographic Details for Blessing Health System eTable 2c. Additional Geographic Details for Haward ARH Regional Medical Center eTable 2d. Additional Geographic Details for Wetaskiwin Hospital (Alberta Health Services) eTable 3. Population Density eTable 4. Detailed Inclusion and Exclusion Criteria eTable 5. Capabilities by Site eTable 6. Process Measures, Home Group eTable 7. Cost and Room and Board Details eTable 8. Patient Safety Measures eTable 9. Utilization Among Intervention Patients, by Home vs Hospital Phase of Care eTable 10. Baseline Patient Characteristics (Home in Less Than 3 Days of Brick-and-Mortar vs Control) eTable 11. Relative Cost of Home Hospital Care (Home in Less Than 3 Days of brick-and-mortar) to brick-and-Mortar Hospital Care eTable 12. Patient Health Care Use (Home in Less Than 3 Days of brick-and-mortar vs Control) eTable 13. Quality, Physical Activity, Functional Status, and Experience (Home in Less Than 3 Days of brick-and-mortar vs. Control) eAppendix 1. Detail Regarding Secondary Outcomes eAppendix 2. Table 1 Missing Data eAppendix 3. Table 4 Missing Data eReference

Supplement 3. Data Sharing Statement

## References

[1] Davis JC, Cromartie J, Farrigan T, Genetin B, Sanders A, Winikoff JB. Rural America at a Glance. 2023 Edition. U.S. Department of Agriculture, Economic Research Service; 2023. doi:10.32747/2023.8134362.ers

[2] Statistics Canada. Population growth in Canada’s rural areas, 2016 to 2021. Population growth in Canada’s rural areas, 2016 to 2021. February 9, 2022. Accessed March 3, 2025. https://www12.statcan.gc.ca/census-recensement/2021/as-sa/98-200-x/2021002/98-200-x2021002-eng.cfm

[3] Parker K, Horowitz JM, Brown A, Fry R, Cohn D, Igielnik R. What Unites and Divides Urban, Suburban and Rural Communities. Pew Research Center; 2018.

[4] The Cecil G. Sheps Center for Health Services Research. Rural hospital closures - Sheps Center. Accessed October 24, 2024. https://www.shepscenter.unc.edu/programs-projects/rural-health/rural-hospital-closures/

[5] Bates DW, Levine DM, Salmasian H, . The safety of inpatient health care. N Engl J Med. 2023;388(2):142-153. doi:10.1056/NEJMsa2206117 36630622

[6] Creditor MC. Hazards of hospitalization of the elderly. Ann Intern Med. 1993;118(3):219-223. doi:10.7326/0003-4819-118-3-199302010-00011 8417639

[7] Johnston KJ, Wen H, Kotwal A, Joynt Maddox KE. Comparing preventable acute care use of rural versus urban Americans: an observational study of national rates during 2008-2017. J Gen Intern Med. 2021;36(12):3728-3736. doi:10.1007/s11606-020-06532-4 33511571 PMC8642477

[8] Joynt KE, Orav EJ, Jha AK. Mortality rates for Medicare beneficiaries admitted to critical access and non-critical access hospitals, 2002-2010. JAMA. 2013;309(13):1379-1387. doi:10.1001/jama.2013.2366 23549583

[9] Joynt KE, Harris Y, Orav EJ, Jha AK. Quality of care and patient outcomes in critical access rural hospitals. JAMA. 2011;306(1):45-52. doi:10.1001/jama.2011.902 21730240 PMC3337777

[10] Truong TT, Siu AL. The evolving practice of hospital at home in the United States. Annu Rev Med. 2024;75:391-399. doi:10.1146/annurev-med-051022-042210 37729030

[11] Arsenault-Lapierre G, Henein M, Gaid D, Le Berre M, Gore G, Vedel I. Hospital-at-home interventions vs in-hospital stay for patients with chronic disease who present to the emergency department: a systematic review and meta-analysis. JAMA Netw Open. 2021;4(6):e2111568. doi:10.1001/jamanetworkopen.2021.11568 34100939 PMC8188269

[12] Levine DM, Pian J, Mahendrakumar K, Patel A, Saenz A, Schnipper JL. Hospital-level care at home for acutely ill adults: a qualitative evaluation of a randomized controlled trial. J Gen Intern Med. 2021;36(7):1965-1973. doi:10.1007/s11606-020-06416-7 33479931 PMC8298744

[13] Leff B, Burton L, Mader SL, . Hospital at home: feasibility and outcomes of a program to provide hospital-level care at home for acutely ill older patients. Ann Intern Med. 2005;143(11):798-808. doi:10.7326/0003-4819-143-11-200512060-00008 16330791

[14] Wang X, Stewart C, Lee G. Patients’ and caregivers’ perceptions of the quality of hospital-at-home service: A scoping review. J Clin Nurs. 2024;33(3):817-838. doi:10.1111/jocn.16906 37817557

[15] Hernandez C, Tukpah AC, Mitchell HM, . Hospital-level care at home for patients with acute respiratory disease: a descriptive analysis. Chest. 2023;163(4):891-901. doi:10.1016/j.chest.2022.11.006 36372302

[16] Levine DM, Paz M, Burke K, . Remote vs in-home physician visits for hospital-level care at home: a randomized clinical trial. JAMA Netw Open. 2022;5(8):e2229067. doi:10.1001/jamanetworkopen.2022.29067 36040741 PMC9428739

[17] Moss CT, Schnipper JL, Levine DM. Caregiver burden in a home hospital versus traditional hospital: A secondary analysis of a randomized controlled trial. J Am Geriatr Soc. 2024;72(1):286-289. doi:10.1111/jgs.18603 37789659

[18] Federman AD, Soones T, DeCherrie LV, Leff B, Siu AL. Association of a bundled hospital-at-home and 30-day postacute transitional care program with clinical outcomes and patient experiences. JAMA Intern Med. 2018;178(8):1033-1040. doi:10.1001/jamainternmed.2018.2562 29946693 PMC6143103

[19] Cryer L, Shannon SB, Van Amsterdam M, Leff B. Costs for ‘hospital at home’ patients were 19 percent lower, with equal or better outcomes compared to similar inpatients. Health Aff (Millwood). 2012;31(6):1237-1243. doi:10.1377/hlthaff.2011.1132 22665835

[20] Levine DM, Ouchi K, Blanchfield B, . Hospital-level care at home for acutely ill adults: a randomized controlled trial. Ann Intern Med. 2020;172(2):77-85. doi:10.7326/M19-0600 31842232

[21] Levine DM, Souza J, Schnipper JL, Tsai TC, Leff B, Landon BE. Acute hospital care at home in the united states: the early national experience. Ann Intern Med. 2024;177(1):109-110. doi:10.7326/M23-2264 38190713 PMC10872234

[22] Adams D, Wolfe AJ, Warren J, . Initial findings from an acute hospital care at home waiver initiative. JAMA Health Forum. 2023;4(11):e233667. doi:10.1001/jamahealthforum.2023.3667 37921747 PMC10625041

[23] Clarke DV, Newsam J, Olson D, Adams D, Wolfe A, Fleisher LA. Acute hospital care at home: the cms waiver experience. July 12, 2021. Accessed July 22, 2022. https://catalyst.nejm.org/doi/full/10.1056/CAT.21.0338

[24] Centers for Medicare & Medicaid Services (CMS). Acute hospital care at home individual waiver only (not a blanket waiver). QualityNet. Accessed August 8, 2021. https://qualitynet.cms.gov/acute-hospital-care-at-home

[25] Raîche M, Hébert R, Dubois MF. PRISMA-7: a case-finding tool to identify older adults with moderate to severe disabilities. Arch Gerontol Geriatr. 2008;47(1):9-18. doi:10.1016/j.archger.2007.06.004 17723247

[26] Galvin JE, Roe CM, Powlishta KK, . The AD8: a brief informant interview to detect dementia. Neurology. 2005;65(4):559-564. doi:10.1212/01.wnl.0000172958.95282.2a 16116116

[27] Kroenke K, Spitzer RL, Williams JBW. The Patient Health Questionnaire-2: validity of a two-item depression screener. Med Care. 2003;41(11):1284-1292. doi:10.1097/01.MLR.0000093487.78664.3C 14583691

[28] Ader DN. Developing the Patient-Reported Outcomes Measurement Information System (PROMIS). Med Care. 2007;45(suppl 1):S1-S2. doi:10.1097/01.mlr.0000260537.45076.74 17443116 PMC2829758

[29] Chew LD, Bradley KA, Boyko EJ. Brief questions to identify patients with inadequate health literacy. Fam Med. 2004;36(8):588-594. 15343421

[30] van Hout B, Janssen MF, Feng YS, . Interim scoring for the EQ-5D-5L: mapping the EQ-5D-5L to EQ-5D-3L value sets. Value Health. 2012;15(5):708-715. doi:10.1016/j.jval.2012.02.008 22867780

[31] Lawton MP, Brody EM. Assessment of older people: self-maintaining and instrumental activities of daily living. Gerontologist. 1969;9(3):179-186. doi:10.1093/geront/9.3_Part_1.179 5349366

[32] van Hees VT, Sabia S, Jones SE, . Estimating sleep parameters using an accelerometer without sleep diary. Sci Rep. 2018;8(1):12975. doi:10.1038/s41598-018-31266-z 30154500 PMC6113241

[33] About the Net Promoter System | Bain & Co. Accessed January 10, 2025. https://www.netpromotersystem.com/about/

[34] Adams C, Walpola R, Schembri AM, Harrison R. The ultimate question? Evaluating the use of Net Promoter Score in healthcare: A systematic review. Health Expect. 2022;25(5):2328-2339. doi:10.1111/hex.13577 35985676 PMC9615049

[35] Krol MW, de Boer D, Delnoij DM, Rademakers JJDJM. The Net Promoter Score–an asset to patient experience surveys? Health Expect. 2015;18(6):3099-3109. doi:10.1111/hex.12297 25345554 PMC5810704

[36] Goodman RA, Posner SF, Huang ES, Parekh AK, Koh HK. Defining and measuring chronic conditions: imperatives for research, policy, program, and practice. Prev Chronic Dis. 2013;10:E66. doi:10.5888/pcd10.120239 23618546 PMC3652713

[37] American Geriatrics Society 2012 Beers Criteria Update Expert Panel. American Geriatrics Society updated Beers Criteria for potentially inappropriate medication use in older adults. J Am Geriatr Soc. 2012;60(4):616-631. doi:10.1111/j.1532-5415.2012.03923.x 22376048 PMC3571677

