# Supplementary material for: Hospital-Level Care at Home for Adults Living in Rural Settings: A Randomized Clinical Trial
Source: JAMA Netw Open. 2025 Dec 1;8(12):e2545712. doi: 10.1001/jamanetworkopen.2025.45712 (PMC12670196; doi:10.1001/jamanetworkopen.2025.45712)
Supplement: Supplement 1. — Trial Protocol [file jamanetwopen-e2545712-s001.pdf]

DETAILED PROTOCOL

Hospital-Level Care at Home for Acutely Ill Adults in Rural Settings:
A Randomized Controlled Trial

Principal Investigator: Dr. David Levine

Sponsor: Thompson Foundation

Updated: **July 2023**

CONTENTS

1.0 BACKGROUND AND SIGNIFICANCE
1.1 HISTORICAL BACKGROUND
1.1.1 Previous clinical studies
1.1.2 Rationale

2.0 SPECIFIC AIMS
2.1 Primary Aim
2.2 Secondary Aims

3.0 SUBJECT SELECTION
3.1 Inclusion
3.2 Exclusion
3.3 Source of subjects and recruitment methods

4.0 SUBJECT ENROLLMENT
4.1 Screening and recruitment
4.2 Methods of enrollment and Procedures for obtaining consent/ Informed Consent
4.3 Treatment Assignment

5.0 STUDY PROCEDURES
5.1 Study visits and measured parameters
5.2 Drugs to be used
5.3 Devices to be used
5.4 Procedures/surgical interventions
5.5 Data to be collected and when the data will be collected
5.5.1 Primary endpoint
5.5.2 Secondary and Exploratory endpoints
5.5.3 Covariates of Interest
5.6 Standard Operating Procedures for problematic situations

6.0 BIOSTATISTICAL ANALYSIS
6.1 Quantitative
6.2 Qualitative

7.0 RISKS AND DISCOMFORTS

8.0 POTENTIAL BENEFITS

9.0 MONITORING AND QUALITY ASSURANCE

10.0 APENDIX

11.0 REFERENCES

1.0 BACKGROUND AND SIGNIFICANCE

1.1 Historical Background

Hospitals are the standard of care for acute illness in the US, but hospital care is expensive and often unsafe, particularly for older individuals.[1] While admitted, 20% suffer delirium,[2] over 5% contract hospital-acquired infections,[3] and most lose functional status that is never regained.[4] Timely access to inpatient care is poor: many hospital wards are typically over 100% capacity, and emergency department waits can be protracted. An acute illness that traditionally requires inpatient hospitalization represents a common critical moment in people's lives. Many people worldwide lack access to acute care, some face intense travel burdens to obtain care, while others simply go without. Even those who may receive their care in a hospital will encounter an environment that may be unsafe and expensive. Maintaining high quality acute care facilities in rural areas is a major burden to health care systems.

1.1.1 Previous Clinical studies

We have designed, implemented, evaluated, and continue improving on home-based acute care (e.g., a "home hospital") for dense urban environments. The home hospital model of care provides in-home acute care to acutely ill patients who would normally be admitted to a traditional hospital. Studies of the home hospital model have demonstrated that a sizeable proportion of acute care can be delivered in the home with equal quality and safety, reduced cost, and improved patient experience.[5,6] In late 2016, we launched a randomized controlled trial (RCT) of home hospital care, the first RCT to be performed in the US context. With this pilot weshowed that hospital-level care could be delivered with similar or better quality and safety but at lower cost.[7] We subsequently completed a larger RCT that corroborated the pilot's findings, additionally demonstrating reduced readmissions for those home hospitalized.[8] Since conclusion of this larger RCT, we have enrolled over 800 patients in home hospital care.

1.1.2 Rationale

About 1 in 5 Americans live in rural areas, totaling nearly 60 million people.[9] Living in a rural area is associated with higher mortality[10] and poor access to health care, with 23% reporting an average 34-minute drive to their nearest hospital.[11] An acute illness that traditionally requires hospitalization represents a common critical moment in nearly every person's life. While some in rural areas face intense travel burdens to obtain acute care, others simply go without. Even those who receive care in a hospital may encounter an environment that is unsafe, of poor quality or experience, and expensive.[11] This may be particularly true of rural hospitals.[12,13] In response to this unacceptable geographic disparity, over the last 2 years, our team has designed and refined the rural home hospital (RHH) model through background research, workflow mapping, prototyping, and rapid cycle testing. We began with interviews and focus groups of clinicians and patients working and living in rural areas (manuscript under review). We conducted a simulation of RHH through a series of "mock admissions" with stable but chronically ill patients in rural Utah who were asked to feign an acute exacerbation. We evaluated the processes, workflows, and technologies involved in RHH and collected qualitative data from the patients, caregivers, and RHH clinical team (manuscript under review). Through the "mock admissions",

we learned how to best operationalize a care plan and interact with a patient both in their home and remotely. Importantly, we heard that patients are accepting and eager to receive care with this model.

Building on the success of these detailed planning exercises, we have taken these design insights into practice through a feasibility study with real patients which is currently underway with the University of Utah Health (protocol 2020P000708). The next phase of this work is to generate evidence that demonstrates the transformative potential of this model. Through a randomized controlled trial, we hope to assess whether home hospitalized patients receive high-quality care that costs less as compared to traditional hospital care in a rural setting. The proposed study will lay the groundwork for disseminating and scaling the RHH innovation across the country in the next three to five years.

The proposed RHH randomized controlled trial will assess whether home hospitalized patients receive high-quality care that costs less as compared to traditional hospital care. Given the success of the urban model, and the adaptation insights gained during the design and feasibility phases of the project, we are cautiously optimistic the study results will be favorable. However, a null result would still provide high-value information to understand the strengths and weaknesses of home-based acute care in rural contexts. Rural health care in the U.S. is already shifting rapidly as hospitals adapt to worsening financial strain, a contextual factor which the COVID-19 pandemic is further exacerbating. The knowledge generated by this study will equip payers, providers, and policy makers with the evidence to make smart strategic decisions for utilizing scarce resources.

2.0 SPECIFIC AIMS

2.1 Primary Aim

Accomplish at least a 10% reduction in hospitalization cost for select hospitalized adults who would normally have been admitted to the hospital in a rural area.

2.2 Secondary Aims

1. Demonstrate rural home hospital care improves days at home, physical activity, and readmission.
    - For example: reduced length of stay, increased daily steps and hours of physical activity per day.
2. Synthesize lessons learned to prepare a dissemination guide for a future multi-site implementation.
    - For example: clinical and administrative data including data collected from the process measures and qualitative interviews with patients and caregivers.
    - Clinician process measures survey with rural home hospital clinicians
    - Qualitative interviews with rural home hospital clinicians.

Please refer to our performance measures below for more details regarding the definitions and sources for each of these measures

3.0 SUBJECT SELECTION

3.1 Inclusion

<u>Patient inclusion criteria:</u>

Patient clinical inclusion criteria:

- >=18 years old
- Any infectious process (e.g., pneumonia, diverticulitis, cellulitis, complicated urinary tract infection)
- Heart failure exacerbation
- Asthma and chronic obstructive pulmonary disease exacerbation
- Atrial fibrillation with rapid ventricular response
- Diabetes and its complications
- Venous thromboembolism
    - This includes a patient who requires therapeutic anticoagulation and concomitant monitoring (thus requiring inpatient status)
- Gout exacerbation
- Chronic kidney disease with volume overload
- Hypertensive urgency
- End of life / desires only medical management
    - This includes a patient who requires acute care for symptom management but declines any surgical intervention. This may include a patient who is about to transition to hospice care, for example, but still has the functional capacity to meet our criteria below. Under these circumstances, we would make sure that various contingencies, including possible transition to hospice care or hospital readmission, are completely understood by patients and caregivers as applicable.

Patient environmental inclusion criteria:

- Lives in a rural area (see definitions in Appendix) that can be served by the RHH team.
- Has capacity to consent to study OR can assent to study and has proxy who can consent (see subject enrollment, below)
- Can identify a potential caregiver who agrees to stay with patient for first 24 hours of admission. Caregiver must be competent to call care team if a problem is evident to her/him. After 24 hours, this caregiver should be available for as-needed spot checks on the patient.
    - This criterion may be waived for highly competent patients at the patient and clinician's discretion.

<u>Patient caregiver inclusion criteria: (</u>not required for patient participation):

- Age >= 18 years old
- Has capacity to consent to study
- Lives within 15 minutes travel time.

<u>Clinician inclusion criteria:</u>

- The rural home hospital clinical team will be identified by the site PI at each study site prior to the start of the study. The site PI will recruit local RNs and/or EMT-Ps, and attending physicians (MD) to deploy and provide rural home hospital care.

- Any member of the rural home hospital clinical team (a clinician providing care in the home) who will be participating in research activities, including the screening and recruitment of patients for the rural home hospital intervention and/or providing care to rural patients that enroll in the intervention.

Sites without continuous monitoring will make amendments to the above inclusion criteria

3.2 Exclusion

<u>Patient exclusion criteria:</u>

Patient exclusion clinical criteria:

- Acute delirium, as determined by the Confusion Assessment Method
- Cannot establish peripheral access by any means
- Secondary condition: active non-melanoma/prostate cancer, end-stage renal disease, acute myocardial infarction, acute cerebral vascular accident, acute hemorrhage (unless part of end of life pathway)
- Primary diagnosis requires multiple or routine administrations of intravenous narcotics for pain control
- Cannot independently ambulate to bedside commode, unless home-based aides are available
- As deemed by on-call MD, patient likely to require any of the following procedures that have not already occurred: computed tomography, magnetic resonance imaging, endoscopic procedure, blood transfusion, cardiac stress test, or surgery (unless these can be coordinated with appropriate facilities during the home hospitalization)
- For pneumonia:
    - Most recent CURB65 > 3: new confusion, BUN > 19mg/dL, respiratory rate>=30/min, systolic blood pressure<90mmHg, Age>=65 (<14% 30-day mortality)[15]
    - Most recent SMRTCO > 2: systolic blood pressure < 90mmHg (2pts), multilobar CXR involvement (1pt), respiratory rate >= 30/min, heart rate >= 125, new confusion, oxygen saturation <= 90% (<10% chance of intensive respiratory or vasopressor support)[16]
    - Absence of clear infiltrate on imaging
    - Cavitary lesion on imaging
    - Pulmonary effusion of unknown etiology
    - O_2 saturation < 90% despite 5L O_2
- For heart failure:
    - Has a left ventricular assist device

  - GWTG-HF[17] (>10% in-hospital mortality) or ADHERE[18] (high risk or intermediate risk 1)*
  - Severe pulmonary hypertension
- For complicated urinary tract infection:
  - Absence of pyuria
  - Most recent qSOFA > 1 (SBP≤100 mmHg, RR≥22, GCS<15 [any AMS]) (if sepsis, >10% mortality)[19]
- For other infection
  - Most recent qSOFA > 1 (SBP≤100 mmHg, RR≥22, GCS<15 [any AMS]) (if sepsis, >10% mortality)[19]
- For COPD
  - BAP-65 score > 3 (BUN>25, altered mental status, HR>109, age>65) (<13% chance in-hospital mortality): exercise caution
- For asthma
  - Peak expiratory flow < 50% of normal: exercise caution
- For diabetes and its complications
  - Requires IV insulin
- For hypertensive urgency
  - Systolic blood pressure > 190 mmHg
  - Evidence of end-organ damage; for example, acute kidney injury, focal neurologic deficits, myocardial infarction
- For atrial fibrillation with rapid ventricular response
  - Likely to require cardioversion
  - New atrial fibrillation with rapid ventricular response
  - Unstable blood pressure, respiratory rate, or oxygenation
  - Despite IV beta and/or calcium channel blockade in the emergency department, HR remains > 125 and SBP remains different than baseline
  - Less than 1 hour of time has elapsed with HR < 125 and SBP similar or higher than baseline
- Home hospital census is full

*GWTG-HF: AHA Get with the Guidelines: SBP, BUN, Na, Age, HR, Black race, COPD
ADHERE: Acute decompensated heart failure national registry: BUN, creatinine, SBP

Patient environmental exclusion criteria:

- Undomiciled
- No working heat (October-April), no working air conditioning if forecast > 80°F (June-September), or no running water
- On methadone requiring daily pickup of medication
- In police custody
- Resides in facility that provides on-site medical care (e.g., skilled nursing facility)
- Domestic violence screen positive[8]

Sites without continuous monitoring will make amendments to the above exclusion criteria

3.3 Source of subjects and recruitment methods

All subjects will be associated with rural hospital sites. This study will have three study sites, two U.S. based sites, and one site based in Canada.

Mass General Brigham will serve as the single-site IRB for the U.S. sites. The two U.S. study sites will be:

- Blessing Health System, IL
- Appalachian Regional Healthcare System, KY

The third study site has received local IRB approval for this study (approval certificate is attached in Insight)

- Alberta Health Services, AB, Canada (Wetaskiwin hospital)

A patient may be considered for RHH care if they are acutely ill at home, present acutely ill to a clinic or emergency department, or if they are admitted and require additional days of acute care ("early transfer"):

- <u>If at home</u>: this patient phenotype is well-known to her/his physician and has an acute illness of highly likely etiology (e.g., heart failure or chronic obstructive pulmonary disease exacerbation). The patient's care team (perhaps primary care or a specialist) will receive a call from the patient describing acute symptoms. The care team will call the RHH clinical coordinator to describe the case. If appropriate, her/his physician will ask the patient if the research assistant/coordinator can approach the patient regarding the rural home hospital study. If the patient agrees, the research assistant/coordinator will call the patient to determine eligibility and introduce the rural home hospital to the patient, and explain randomization If the patient is eligible and interested in participating, the RHH MD or RHH Advanced Practice Provider (APP) will make the decision to admit and will consent the patient. If randomized home, the RHH nurse/paramedic will meet the patient in their home.
- <u>If presenting acutely ill to a clinic</u>: after appropriate assessment by the clinic care team, and if the team believes the patient requires admission, the clinic care team will call the RHH research assistant/coordinator to describe the case. If appropriate, her/his clinic care team will ask the patient if the research assistant/coordinator can approach the patient regarding the rural home hospital study. If the patient agrees, the research assistant/coordinator will call the patient to determine eligibility introduce the study to the patient and explain randomization. If the patient is eligible and interested in participating, the RHH MD or RHH Advanced Practice Provider (APP) will make the decision to admit and consent the patient. If randomized home, the patient will be transported home in an appropriate transport (personal car, chair car, or ambulance). If randomized home, the RHH nurse/paramedic will meet the patient in their home.
- <u>If presenting acutely ill to an emergency department</u>: after appropriate assessment by the emergency department team, and if the team believes the patient requires admission, the emergency department care team will ask the patient if the research assistant/coordinator can approach the patient regarding the rural home hospital study. If appropriate, the

research assistant/coordinator will call the patient to determine eligibility, introduce the rural home hospital to the patient and explain randomization. If the patient is eligible and interested in participating, the RHH MD or RHH Advanced Practice Provider (APP) will make the final decision to admit and consent the patient. If randomized home, the patient will be transported home in an appropriate transport (personal car, chair car, or ambulance). If randomized home, the RHH nurse/paramedic will meet the patient in their home.

- If admitted and requiring additional days of acute care (“early transfer”): an example ideal patient for this setting is a patient with a heart failure exacerbation requiring additional days of IV diuresis and close monitoring including daily electrolyte and telemetry monitoring. After appropriate assessment by the hospital care team and if the care team believes the patient requires additional days of acute care but could be care for with rural home hospital, the hospital care team will ask the patient if the research assistant/coordinator can approach the patient regarding the rural home hospital study. If appropriate, the research assistant/coordinator will determine eligibility, introduce the study to the patient, and explain randomization. The hospital care team, research assistant/coordinator, and RHH MD or RHH Advanced Practice Provider (APP) will determine if the patient is eligible for RHH and if the patient and all stakeholders are in agreement with RHH, the patient will be enrolled and consented to RHH by the RHH MD or RHH Advanced Practice Provider (APP). If randomized home, the patient will be transported home in an appropriate transport (personal car, chair car, or ambulance). If randomized home, the RHH nurse/paramedic will meet the patient in their home.

4.0 SUBJECT ENROLLMENT

4.1 Screening and recruitment

The project’s recruitment, screening/admission, and deployment model is illustrated in Figure 2. Patient recruitment may begin at the emergency department, clinic, home, or as an early transfer from an inpatient hospitalization. Patients will be randomly allocated to rural home hospital care (intervention) or traditional hospital care (control). Please see section 4.3 for more details on treatment assignment.

Patients:

- Acutely ill patient is screened and recruited as above (section 3.3).
- 50 patients at each study site
- If the patient meets eligibility and agrees to enroll in the study the research assistant will contact the on-call rural home hospital MD or Advanced Practice Provider (APP) who will assess the patient’s clinical criteria by EHR review.

Caregivers:

- 50 caregivers at each study site
- Caregivers will be pre-screened by a research assistant/coordinator for inclusion criteria. If the participant agrees, the research assistant/coordinator will introduce the study to the caregiver, explain randomization, and answer initial questions.

Clinicians

- 12 clinicians at Blessing Health and ARH
- 40 clinicians at AHS
- Members of the rural home hospital clinical team will be identified by the site PI at each study site prior to the start of the study.

4.2 Methods of enrollment and Procedures for obtaining consent/ Informed Consent

Patients:

- If the participant has interest in the program and meets inclusion/exclusion criteria, the research assistant/coordinator will assist in the informed consent process by reviewing the consent form with the patient caregiver. The RHH MD or Advanced Practice Provider (APP) will then further discuss the study and consent form with the patient and answer any questions. The MD or Advanced Practice Provider (APP) (either in person**,** over the phone or via a live video visit) will obtain written informed consent or electronic informed consent [through REDCap (Research Electronic Data Capture, Vanderbilt University, Nashville, TN), which is fully HIPAA-compliant secure web application] of the participant to enroll in the RHH study.
- The RHH MD or APP will also obtain consent from the patient to receive text messages from the study, the consenting MD/APP will present the warning language on the text message consent form during the consent process.
- If the patient is deemed without capacity, any of the following individuals (listed in general order of preference) may give consent provided they are on-site and able to sign the informed consent (the patient will assent):
    - court appointed guardian with specific authority to consent to participation in research or authority to make health care decisions for a class of diagnostic and therapeutic decisions inclusive of the proposed research;
    - health care proxy/person with durable power of attorney with specific authority for making health care decisions inclusive of the proposed research; or
    - spouse, adult child, or other close family member who knows the subject well and has been involved in their care.
- In the case of surrogate consent, a patient must be returning to their prior level of supervision.
    - For patients who cannot consent, their home situation must be set up with the same prior level of supervision that existed prior to enrollment. For example, if a patient had 24/7 care prior to the admission, then upon admission, they would need to have that same 24/7 care in place.
- Subjects who do not speak English will use a short-form consent form with the assistance of a certified interpreter.

- The RHH MD or Advanced Practice Provider (APP) will make the final decision to admit the patient to home hospital.

Caregivers:

- Caregivers will be pre-screened by a research assistant/coordinator for inclusion criteria. If the participant agrees, the research assistant/coordinator will further describe the program and answer initial questions. If the caregiver expresses interest and meets inclusion criteria the research assistant/coordinator will assist in the informed consent process by reviewing the consent form with the caregiver. The RHH MD or Advanced Practice Provider (APP) will then discuss the study and consent form with the caregiver, answer any questions, and obtain written informed consent or electronic informed consent [through REDCap (Research Electronic Data Capture, Vanderbilt University, Nashville, TN), which is fully HIPAA-compliant secure web application].
- The RHH MD or APP will also obtain consent from the caregivers to receive text messages from the study, the consenting MD/APP will present the warning language on the text message consent form during the consent process.
-

Clinicians:

- Members of the rural home hospital clinical team will be identified by the site PI at each study site prior to the start of the study. The site PI will approach each clinician and ask if they are interested in participating in research activities. If the clinician agrees, the research assistant/coordinator will discuss the purpose of the study with them (including what data we will be collecting from rural home hospital clinicians and why) and invite them to participate. If the clinician agrees to enroll, the research assistant/coordinator will assist in the informed consent process by reviewing the consent form with the clinician. The site coordinator or the RHH MD or RHH APP will then discuss the study and consent form with the clinician, answer any questions, and obtain written informed consent or electronic informed consent [through REDCap (Research Electronic Data Capture, Vanderbilt University, Nashville, TN), which is fully HIPAA-compliant secure web application] from clinician participants.
- The site coordinator, RHH MD, or APP will also obtain consent from the RHH Clinician to receive text messages from the study, the consenting MD/APP will present the warning language on the text message consent form during the consent process.

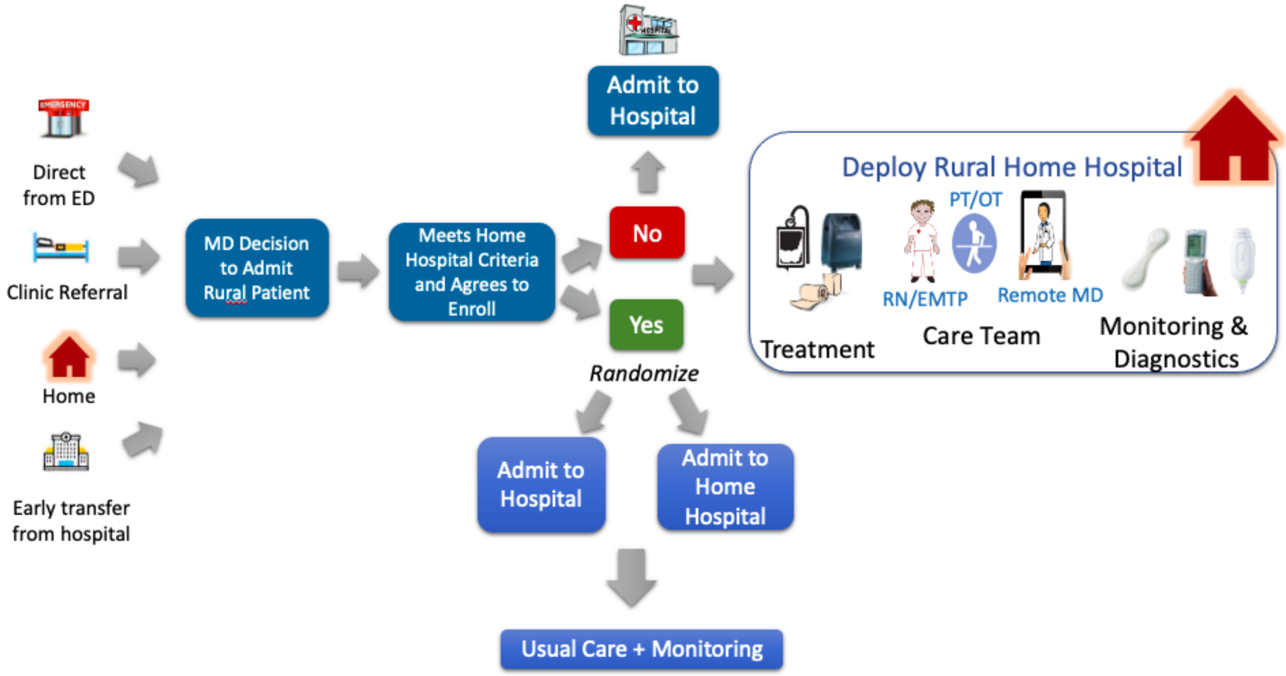

Figure 2

4.3 Treatment Assignment

- If the participant consents, the research assistant will proceed to randomization via concealed envelopes with
  - Concealed allocation;
  - Stratified on diagnosis:
    - infection,
    - heart failure,
    - chronic obstructive pulmonary disease or asthma, and
    - other diagnosis;
  - Randomly selected block sizes between 4 and 6;
  - Use randomization to determine arm.
- If patient/caregiver does not consent, the research assistant will ask the patient's permission to collect covariates, functional status, and a quality-of-life measure to determine generalizability of enrollment.
- Participants randomized to the control group will receive usual care at the hospital, from an attending general internist.

5.0 STUDY PROCEDURES

5.1 Study visits and measured parameters

Standard admission group:

An activity tracker (Vitalpatch-discussed under section 5.3) will be placed on all subjects in the control group during admission only. Subjects in the control group will receive usual care at the hospital, from an attending general internist.

Rural home hospitalization group (intervention group):

Each day, the RHH clinical team will make in-person and/or virtual visits to the patient's home. These are personnel who have been trained in home health and carry out home health as per the usual care they deliver. They have received additional training in RHH. The RHH module offers most of the same medical components that are standard of care in an acute care hospital (**Table below**). The typical staff (MD, RN, or paramedic), diagnostics (blood tests, vital signs, telemetry), intravenous therapy, and oxygen/nebulizer therapies will all be available.

RHH improves upon the components of a typical ward's standard of care in several ways:

- Minimally invasive continuous vital signs, telemetry, and activity tracking
- 24/7 clinician video visits;
- Ambulatory/portable infusion pumps that can be worn on the hip;

Component	Module
Personnel	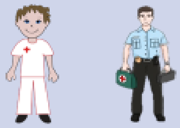 **Nurse/Paramedic** • 2 daily visits (1 in-person; 1 virtual) • Medication administration • IV access management • Education + 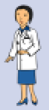 **Physician** • 1 daily interaction • Medical decision making
Diagnostics	Point of care: BMP, Hgb/Hct, PT/INR, ultrasound, x-ray, PFTs, ECG
Pharmacy	Any inpatient medication, most any route
Equipment	Ambulatory infusion pump, peripheral IV, oxygen concentrator
Communication	Telepresence/virtual visits
Monitoring	Wireless remote vital signs, telemetry, activity tracking

Table 1

As denoted in the table above, the physician will make at least one daily virtual visit to the patient's home, facilitated by the nurse/paramedic. At the discretion of the physician and nurse/paramedic, the physician can make additional virtual visits should the patient's clinical condition require it. Similarly, the nurse/paramedic will make at least two daily visits to the patient's home. At the discretion of the physician and nurse/paramedic, the nurse/paramedic can make additional visits should the patient's clinical condition require it. This will be an ongoing twice daily discussion among clinicians, patient, and caregivers, as applicable. A patient or

clinician can initiate a video visit at any time. As denoted in the table above, the RHH team uses a suite of communication software to communicate with the patient and among team members. The nurse or paramedic will provide technology training to the patient (as needed) at admission and the rural home hospital team will be available to provide any technical assistance to subjects as needed.

For patients with chronic/stable dementia, they will receive mental status assessments (eg, Confusion Assessment Method) by the home hospital RN/paramedic at each visit (at least 2 daily). As is standard of care, we will take measures to avoid delirium: open blinds to allow light in during the day, minimize environmental noise, encourage typical routines to optimize eating and sleeping, and reassess medication list for culprit medications.

Clinical parameters measured will be at the discretion of the physician and nurse/paramedic, who treat the participant following evidence-based practice guidelines, just as in the usual care setting. Monitoring will be performed by RHH clinicians. The clinical home hospital team manually checks vital signs at least daily. If deemed necessary based on a patient's acuity, the home hospital team can visit additional times. At all times, the patient and care team have 24/7 access to the RHH attending physician.

On top of this "on-the-ground" team, the home hospital attending is notified with an alarm should there be delayed data transfer. Such an alarm would cause the attending to contact the patient as deemed clinically appropriate.

Please see below for all collected data points.

Documentation by RN/paramedic and MD will occur via digital intake notes, daily progress notes, and discharge notes that will be compiled (on a secure shared drive) and uploaded as a scanned document to the patient's chart upon discharge.

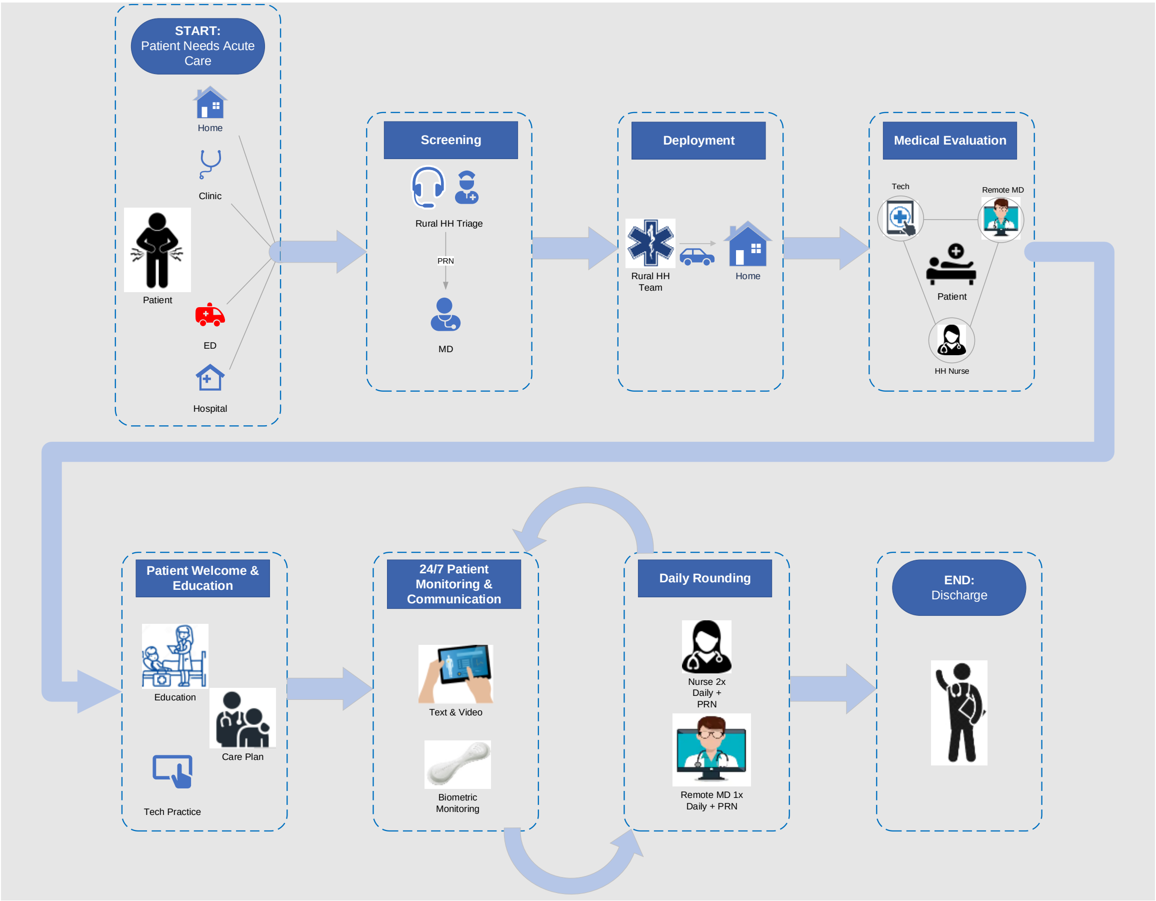

Figure 1 RHH flow. Note a patient may **enter** this flow from their home, a clinic, the emergency department, or the hospital floor. The patient is triaged by the RHH nurse with as needed assistance of the RHH MD. The RHH nurse/paramedic deploys in the patient's home, facilitates a medical evaluation by a remote MD, welcomes and educates the patient, and sets up monitoring and communication technology. The nurse rounds at least once daily in person, facilitating an interaction with the RHH MD. Finally, the patient is discharged using the same criteria for discharge as would be used in a traditional hospital (ability to perform activities of daily living given supports in the home).

5.2 Drugs to be used

Only medications used in the usual care of hospitalized patients will be used. For example, a patient with pneumonia might receive guideline-based antibiotics with ceftriaxone and azithromycin.

5.3 Devices to be used

- Home hospital employs standard devices during the usual care of hospitalized patients. For example, a nebulizer machine.
- Telehealth platform: Biofourmis AI platform, biovitals® Hospital@Home. Biofourmis has completed their cybersecurity assessment and has been vetted by the Partners data sharing committee. The Biofourmis tablet serves as the communication hub and facilitates videoconferencing between the patient and physcian/RN/EMT-P and it captures biometric data.
- Home hospital additionally employs
    - Vital signs monitoring patch: Vital Connect VitalPatch (FDA cleared application/use)
        - The patch will be placed on subjects in both groups, it includes a built-in activity tracker (we will use the activity tracker for control group subjects during hospital admission)
    - Ambulatory infusion pump: Smiths Medical CADD Solis VIP (FDA cleared application/use)
    - Point of care diagnostic meter: Abbott iSTAT (FDA cleared application/use) or Siemens EPOC (FDA cleared application/use)
    - Digital stethoscope (Eko; FDA cleared application/use)
    - Point of care ultrasound (Philips Lumify; FDA cleared application/use)

5.4 Procedures/surgical interventions

Home hospital employs the same standard procedures used during the usual care of hospitalized patients. For example, peripheral venipuncture. Need for surgery is a relative exclusion criterion.

5.5 Data to be collected and when the data will be collected

For both groups we will administer a survey to patients on admission, at discharge, and 30-days post-discharge. For both groups, we will administer a survey to caregivers at admission and 30-days post-discharge.

For the intervention arm only, within 30-days of discharge we will conduct qualitative interviews with the patient and their caregiver (if their caregiver consented to participate in the study, caregiver participation is not required for patient enrollment in this study) (please see more details below).

For the intervention arm, we will ask the rural home hospital MD to complete the MD AM Report each morning and the RHH RN/EMT-P to complete the Nursing visit form after each patient visit. Twice a week we will send the RHH clinicians the Clinician process measures survey. Post-discharge we will conduct qualitative interviews with rural home hospital clinicians only (please see more details below).

For both groups:

Clinical and administrative data will be extracted from electronic sources (e.g., enterprise data warehouse, electronic health record (EHR) at study sites). Cost data for the hospitalization and post-discharge period will be obtained from study site financial personnel using direct cost accounting methods. We will also collect information from patients and caregivers using IRB approved surveys and standardized questionnaires (see below). All data will be collected by trained study staff and inputted on a Partners encrypted shared drive. Data collected from filed research collection (including patient surveys) will be stored in REDCap (Research Electronic Data Capture, Vanderbilt University, Nashville, TN), which is fully HIPAA-compliant secure web application.

- All data collected at Alberta Health Services (Wetaskiwin Hospital) will also be stored on MGB REDCap

We will utilize the REDCap+Twillio module for texting. We will use this system to send out text reminders to study subjects to complete REDCap surveys. All the participant data is stored in REDCap (names, emails, phone numbers). Twilio is used to send the text + survey link. When the respondent touches the link, their browser opens on their smart phone and they complete the survey in the secure website. No PHI or PII will be included in the text of the text message. Message text is defined by the study team and approved by the IRB and study subjects will provide written or electronic consent (through REDCap) to receive communication via text message.

Patient and caregiver surveys may be administered in-person, by email, by phone, text message (if patient/caregiver has consented to receiving text messages) (REDCap +Twilio module for texting) or by paper mail. Clinician surveys will be emailed or sent by text message to clinician subjects through REDCap.

We have attached the following surveys (paper version) to this protocol submission:
    - Caregiver admission survey_PAPERSURVEY_Feb2022
    - Caregiver post-discharge survey_PAPERSURVEY_Feb2022
    - Patient discharge survey_PAPERSURVEY_Feb2022
    - Patient post-discharge survey_PAPERSURVEY_Feb2022

The remaining patient and clinician surveys will be electronic (REDCap):
- Patient admission survey
- All clinician forms and surveys
    - MD AM report
    - Nursing visit form
    - Clinician process measures survey

Patient surveys:

- In-person: research assistant/coordinator may administer the patient survey in-person.
- Email: the surveys will be sent by email through REDCap

- Text message: the surveys will be sent by text message through REDCap (REDCap + Twilio module)
- Phone: research assistant/coordinator will administer the paper survey over the phone and input the data into REDCap
- Paper mail: the paper survey will be sent by mail through USPS to the subject. We will also include a pre-addressed/stamped envelope for participants to mail their completed survey back to the study team.

Caregiver surveys:

- In-person: research assistant/coordinator may administer the patient survey in-person.
- Email: the surveys will be sent by email through REDCap
- Text message: the surveys will be sent by text message through REDCap (REDCap + Twilio module)
- Phone: research assistant/coordinator will administer the paper survey over the phone and input the data into REDCap
- Paper mail: the paper survey will be sent by mail through USPS to the subject. We will also include a pre-addressed/stamped envelope for participants to mail their completed survey back to the study team.

Clinician surveys

- E-mail: surveys will be emailed to clinician subjects through REDCap
- Text message: the surveys will be sent by text message through REDCap (REDCap + Twilio module)

5.5.1 Primary endpoint

Measure	Source	Day(s) Obtained
Total cost, hospitalization (published as percent change given sensitivity of these data)	Intervention arm: Home hospital accounting<br>Control arm: hospital accounting system	Discharge

5.5.2 Secondary endpoints

Measure	Source	Day(s) Obtained
Total cost, discharge to 30-days post discharge (published as percent change given sensitivity of these data)	Hospital accounting system	30 days after discharge
Unplanned readmission within 30-days of discharge, %	EHR and patient report	30 days after discharge
Days at home (the number of days spent at home from the day of discharge to 30-days later)	EHR and patient report	30 days after discharge
Percent of day lying down	Biometric monitor	Each day of

		admission

5.5.3 Exploratory endpoints

Health Care Utilization

Measure	Source	Day(s) Obtained
Length of stay, days	RA via MD/RN for home patients; RA EHR review for inpatients	Discharge
IV medication, days	As above.	Daily
Intravenous fluids, days	RA via EHR / administrative data	Daily
Intravenous diuretics, days	RA via EHR/ administrative data	Daily
Intravenous antibiotics, days	RA via EHR/ administrative data	Daily
Oxygen requirement, days	RA via EHR / administrative data	Daily
Nebulizer treatment, days	RA via EHR / administrative data	Daily
Imaging, %	RA via EHR / administrative data	Daily, +30
Lab Orders, #	As above.	Daily, +30
MD sessions, # of notes	As above.	Daily, +30
Consultant sessions, # of notes	As above.	Daily, +30
PT/OT sessions, # of notes	As above.	Daily, +30
Disposition (routine, SNF, home health, other)	RA via MD/RN for home patients; RA EHR review for inpatients/ administrative data	Discharge
Follow up with patient's PCP within 14 days, y/n	RA via pt phone call, cross checked with EHR data	+30
SNF utilization, days	As above.	+30
Home health utilization, days	As above.	+30
Unplanned readmission(s) after index, # and y/n + date	As above.	+30
ED observation stay(s), # and y/n + date	As above.	+30
ED visit(s), # and y/n + date	As above.	+30

RA: Research assistant

Safety

Measure	Source	Day(s) Obtained
Fall	RA via MD/RN for home patients; MD EHR review for inpatients	Daily
Delirium	RNs in home and inpatient settings calculate the CAM (standard of care in both). RA will obtain the home patient CAM via the home RN; RA will obtain the inpatient CAM via EHR.	Daily
DVT/PE	RA via MD/RN for home patients; MD EHR review for inpatients	Daily
New pressure ulcer	As above.	Daily
Thrombophlebitis at peripheral IV site	As above.	Daily
Hospital Acquired Condition		
CAUTI	As above.	Daily
Clostridium difficile	As above.	Daily

MRSA	As above.	Daily
New arrhythmia	As above.	Daily
Hypokalemia	As above.	Daily
Acute kidney injury	As above.	Daily
Medication error	As above.	Daily
Unanticipated mortality	As above.	Daily
Loss of consciousness	As above.	Daily
Transfer back to hospital[a]	RA via MD/RN	Discharge
Unplanned mortality during admission	RA via MD/RN for home patients; MD EHR review for inpatients	Discharge
Unplanned 30-day mortality	RA via pt/caregiver call	+30

[a]: intervention arm only

Quality: High-Value Care

Measure	Source	Day(s) Obtained
Pain management	RA via MD/RN for home patients; RA EHR review for inpatients	Daily
Hours of sleep per day	RA via activity tracker	Daily
Hours of sleep per night	RA via activity tracker	Daily
Hours of activity per day	RA via activity tracker	Daily
Hours of activity per night	RA via activity tracker	Daily
Hours of sitting upright per day	RA via activity tracker	Daily
Hours of sitting upright per night	RA via activity tracker	Daily
Daily steps	RA via activity tracker	Daily

Quality: Low-Value Care

Measure	Source	Day(s) Obtained
Use of inappropriate medications in the elderly	RA via EHR/MAR/administrative data	Daily
Use of foley catheter	RA via MD/RN for home patients; EHR review for inpatients/administrative data	Daily
Use of restraints	RA via MD/RN for home patients; EHR review for inpatients/administrative data	Daily

Patient Functional Status and Quality of Life

Measure	Source	Day(s) Obtained
EuroQol -5D-5L	RA via patient	Admission, discharge, +30
SF-1	RA via patient	admission, discharge, +30
Activities of daily living	RA via patient	admission, discharge, +30
Instrumental activities of daily living	RA via patient	admission, discharge, +30
Patient Health Questionnaire-2	RA via patient	Admission, discharge, +30
PROMIS Emotional Support Short Form 4a	RA via patient	Admission, discharge, +30

Patient and Family Experience

Measure	Source	Day(s) Obtained
Picker Experience Questionnaire	RA via patient	Within 30 days of discharge
Global satisfaction	RA via patient	Within 30 days of discharge
Recommend care	RA via patient	Within 30 days of discharge
Qualitative interviews	See Below	Within 30 days of discharge
Caregiver burden (Zarit)	RA via caregiver	Admission, Within 30 days of discharge

Process Measures

Measure	Source	Day(s) Obtained
Number of RN visits, in-person	RA via RN/EHR	Daily
Number of RN visits, virtual	RA via RN/EHR	Daily
Number of RN visits, total	RA via RN/EHR	Daily
RN travel time	RN	Daily
Failed connectivity, %	RN	Daily
Number of “on call” MD interactions (video or phone)	RA via MD	Daily
Duration of 1st RN visit	RA via RN	Daily
Duration of subsequent RN visit, in-person	RA via RN	Daily
Duration of subsequent RN visit, virtual	RA via RN	Daily
Other metrics captured on the clinician process survey and nursing visit form	RA via RN	Daily
Insufficient handoff	RA via RN	Daily
Documentation error	RA via RN	Daily
Equipment malfunction	RA via RN	Daily

Covariates of Interest

Measure	Source	Day(s) Obtained
Age	RA via EHR[a]	Admission
Gender	RA via EHR	Admission
Race/ethnicity	RA via patient	Admission
Primary language	RA via EHR	Admission
Health insurance status, public/private/none	RA via EHR	Admission
BMI	RA via EHR	Admission
Comorbidities, type and #	RA via EHR and H&P	Admission
Partner status	RA via patient	Admission
Education	RA via patient	Admission
Zip code	RA via EHR	Admission
Employment	RA via H&P	Admission
Smoking status	RA via H&P	Admission
Medications used as outpatient, #	RA via EHR	Admission
DNR/I code status	RA via H&P	Admission
Lives alone	RA via patient	Admission
Home health aide prior to admission	RA via patient	Admission
Elective and urgent admissions in the	RA via EHR and	Admission

previous year, #	patient	
ED visits in the previous 6 months, #	RA via EHR and patient	Admission
Interqual disease-specific leveling	MD	Admission
PRISMA-7	RA via patient	Admission
Eight-item Interview to Differentiate Aging and Dementia	RA via patient or proxy	Admission
Would you be surprised if this patient died in the next year?	RA via MD	Admission
BRIEF health literacy screening tool	RA via patient	Admission
Readmission risk score on discharge (HOSPITAL)	RA via EHR	Discharge
Admitting diagnosis	RA via EHR/MD	Admission
Admission source	RA via EHR/MD	Admission
COVID case count on day of admission	RA via EHR	Admission
Degree of rurality	2010 U.S. Census data, U.S RUCA codes/data files, population density, zip codes	Admission

[a]: Note that RA will recheck covariates after MD H&P completed to ensure comorbidities and other covariates correctly captured

The following data will be collected from intervention arm subjects only (patients, caregivers, and clinicians)

Measure	Data sources	Day(s) Obtained
RHH admission, daily care, and discharge processes accomplished.	Clinician process measures survey	Twice a week
Perceived acceptability of RHH care	Post-discharge semi-structured interviews with RHH clinicians, RHH patients, and caregivers (separate interview guides are attached)	Patients: within 1 month of discharge<br><br>Clinicians: Within 60 days after patient enrollment ends at site
Perceived safety, quality of care, caregiver burden	Post-discharge semi-structured interviews with RHH clinicians, RHH patients, caregivers (separate interview guides are attached)	Patients: within 1 month of discharge<br><br>Clinicians: Within 60 days after patient enrollment ends at site

Video recordings (intervention group only):

- We will video record some of the telemedicine calls between RHH clinicians and rural home hospitalized patients.

- The video recordings will be for internal quality assurance purposes only.
- The video recordings will not be disseminated in any way (not shared externally)
- We have attached the patient interaction monitoring protocol which provides more detail on this process: “ Site Study Team Protocol- Patient Interaction Monitoring”
-

5.5 Standard Operating Procedures for problematic situations

Any subjects presenting with problematic situations will be immediately transferred to the most appropriate hospital and formally withdrawn from the study

Clinically Emergent Patient Condition

Should a matter be emergent, then 9-1-1 will be called and the patient will be brought to the hospital immediately. An example of an emergent patient condition is severe new-onset shortness of breath.

Clinically Urgent Patient Condition

Should a matter be urgent, the patient and/or nurse/paramedic and/or physician may choose to communicate via phone or video (either of the 3 persons can initiate either medium). If this is unsuccessful, the nurse/paramedic will attempt to visit the patient in their home. If there is no way to rectify the situation, then the patient will be transported to the hospital. An example of an urgent situation is new-onset non-severe pain.

Unsafe Home Situation

Should an unsafe home situation be discovered during a home hospital admission, the home hospital team will assess if said situation poses a harm or threat to either the participant or the home hospital personnel. If it does, and after an attempt to rectify said situation, the situation persists, then the home hospital team will end the study and transport the patient to the hospital. An example of an unsafe home situations includes lack of basic sanitation.

Intoxication

Should intoxication occur in the home of a participant, the home hospital team will assess if said intoxication poses a harm or threat to either the participant or the home hospital personnel. If it does, and after an attempt to rectify said intoxication, the situation persists, then the home hospital team will end the study and transfer the patient to the hospital.

Neglect or child abuse

Should neglect or child abuse be observed, the home hospital team will act as mandatory reporters. The home hospital team, in coordination with the hotline team, will assess if said neglect or child abuse poses an immediate harm or threat to either the participant, children in the home, or the home hospital personnel. If it does, then the home hospital team will end the study and transport the patient to the hospital.

Refusal of care

Should an enrolled subject refuse standard care at their home despite a clear discussion with the home hospital team, then the subject will be transferred back to the treating hospital and formally withdrawn from the study.

6.0 BIOSTATISTICAL ANALYSIS

6.1 Quantitative

We will present descriptive data with counts and percentages, means and 95% CIs, or medians and interquartile ranges, as appropriate.

The primary outcome (hospitalization cost) will be analyzed between arms, adjusted for site as a fixed effect, which accounts for difference across the three sites. . However, we will present cost data as the percentage of change in mean costs from control (rather than absolute difference) because of the sensitive nature of these data. The primary outcome will be assessed using a generalized linear model assuming a gamma distribution with a log link, given the skewed nature of cost, with treatment arm (control arm as the reference group) and site as fixed effects. The exponential of the treatment effect in this gamma regression model exactly equals the effect of interest (the percentage change from control). Patient characteristics should balance out across the two arms due to randomization, but if not, in the gamma regression, we will adjust for any of the follow patient characteristics that differ across arms: sex, age (continuous), education, and comorbid condition count (continuous). We *a priori* plan to also adjust for COVID case burden at each hospital only if COVID impacted home hospital operations. If the degree of rurality was different between patients at different sites, we *a priori* plan to adjust for degree of rurality.

We will use this same multivariable regression approach for secondary outcomes, using logistic regression for binary outcomes (e.g., readmissions) and negative binomial regression for count outcomes such as days at home, and non-transformed linear regression for normally distributed continuous outcomes (e.g., days at home), as appropriate. Given the multiple comparisons, we will use the Benjamini-Hochberg method for multiple comparisons for the secondary outcomes, with 0.2 as a prespecified false discovery rate.

We *a priori* plan to perform a subgroup analysis that does not involve any extreme outlier participants (upper quartile plus 3 times the interquartile range) in either study arm, should this occur. We also *a priori* plan subgroup analyses by diagnosis, by age group, and by daily activity level. We may choose to employ non-parametric tests of significance should our data be nonparametric. We *a priori* plan to perform a subgroup analysis between continuous monitoring and intermittent monitoring sites. We *a priori* plan to perform a subgroup analysis by admission source ("from ED" vs "from ward" vs "from home"). We also plan to perform a secondary a priori subgroup analysis of US vs Canada.

Power analysis

A priori (based on data from our previous randomized controlled trial of urban home hospital care), we expect the mean cost to be mean cost on intervention to be 40% lower than in control (leading to a ratio of mean costs in treatment to control to be at most .6). Thus, for our power calculation, we want to be able to detect that the exponential of the treatment effect in our

gamma regression to be 0.6 or lower., We will require 132 patients total (66 in each arm, and 44 total per site) to detect a 40% relative decrease in mean direct cost in home versus hospital (power: 80%, alpha: 0.05, two-tailed) using the Wald test for the treatment effect from a gamma regression model for costs discussed above. In this power calculation, we assume the over-dispersion parameter for the gamma distribution variance is 1.2 (as seen from our preliminary data). Although we hope to recruit 75 patients in each arm, 66 patients will give us more than adequate power (80%) to detect the clinically expected difference.

Secondary outcomes require slightly larger samples sizes at 80% power (with Type 1 error of 1%, approximately the type 1 error when using the Benjamini-Hochberg method as noted above). For Cost, discharge to 30-days, using a gamma regression model similar to total costs, with 66 patients in each arm, we have 80% to see an expected 65% reduction in the mean for intervention to control (we assume the over-dispersion parameter for the gamma distribution variance is 2.8 as seen from our preliminary data). For number of hours per day laying down (equivalently, % of day Lying down), using a negative binomial regression model with a logistic link with treatment and site effects, with 66 patients in each arm, we have over 90% to see an expected 65% reduction in the number of hours lying down for intervention to control (we assume the over-dispersion parameter for the negative binomial distribution is 1.4 as seen from our preliminary data). For percent with 30-day readmission, using logistic regression model with treatment and site effects, with 66 patients in each arm, we have 80% to detect on odds ratio of 0.25 for intervention on control (assuming the percent 30 day readmission in controls is 23% as seen in preliminary data). For the number of days at home, using a log-normal distribution with treatment and site effects, with 66 patients in each arm, we have over 80% to see an expected 65% increase in the number days at home for control to intervention (from 29 days to 48, days assuming the over-dispersion parameter for the negative binomial distribution is 1.4).

6.2 Qualitative

We will conduct semi-structured qualitative interviews (post-discharge interview guides) with each member of the RHH clinical care team and every patient and their caregiver 30 days after discharge. There is one post-discharge interview guide for clinicians and another post-discharge interview guide for patients and caregivers. The interviews will ask clinicians to reflect on their experience caring for home hospitalized patients, including their perceptions on quality and safety of care and comfort level. Clinicians will also be asked about their thoughts on home hospital technology and processes and their experience working with other clinical team members. The interviews with patients and caregivers will ask them to reflect on their experience being home hospitalized, their comfort level, and their perceptions on quality and safety of care provided. Patients and caregivers will also be asked to reflect on their experience with home hospital technology and their experience with the home hospital care team. All participants will be asked if they have any suggestions for improving rural home hospital.

The semi-structured interviews will be recorded, transcribed, and then analyzed using NVivo qualitative analysis software. We will audio record interviews using QuickTime on a Partners encrypted laptop and send them for transcription to a HIPAA-compliant transcription service (TranscribeMe).

Qualitative data will be analyzed using template analysis; two researchers will code the data independently. An initial codebook will be developed using deductive codes derived from the interview questions and the researchers' prior experience with rural home hospitals. Following initial coding, additional codes may be identified and added to the codebook. Inter-coder agreement will be assessed between the two coders and all inconsistencies will be noted, discussed, and resolved by consensus. Themes and recurrent patterns will be identified with any differences resolved by consensus. We will also use these data to improve home hospital operations.

7.0 RISKS AND DISCOMFORTS

Uncommon: Complications of surgical and non-surgical procedures

Standard hospital procedures carry small risks and discomforts. For example, venipuncture can be painful and can lead to thrombophlebitis, but this is uncommon and readily rectifiable.

Uncommon: Device complications/malfunctions

IV pumps, vital signs monitoring patches, and the other devices used during home hospital uncommonly have malfunctions.

Uncommon: Radiation risks

Patients can receive radiography while admitted to home hospital, much the same as when they are admitted in the hospital. We do not anticipate that home hospital will in any way change the prevailing risk of radiation.

Common (but unchanged from usual care): Drug side effects and toxicities

Drug side effects and toxicities do occur during inpatient medicine, despite following best practices and evidence based medicine. We do not anticipate that home hospital will in any way change the prevailing rate of drug side effects and toxicities compared to usual care. For example, acute kidney injury can occur in patients receiving antibiotics, even when correct dosing occurs. Home hospital is equipped to monitor and respond to the common drug side effects and toxicities much the same as the standard of care (monitoring, diagnostics, fluids, etc).

Common (but improved from usual care): Psychosocial risks

Admission to the hospital can be a psychosocially difficult event, particularly for a senior. We believe home hospital will alleviate these typical risks.

8.0 POTENTIAL BENEFITS

Patients (home hospitalization group):

- Remain in their home despite acute illness
    - Eat their own culturally concordant food
    - Sleep in familiar surroundings
    - Maximize time and interactions with their family and friends
- Improved health outcomes (we anticipate these from previous literature)
    - Reduced length of stay

    - Reduced complications while admitted
        - Less delirium
        - Fewer falls
        - Fewer health care associated infections
        - Decreases reduction in functional status
    - Improved patient experience
- Clinical improvements to the standard of care
    - Minimally invasive continuous vital signs, telemetry, and activity tracking;
    - On-demand 24/7 clinician virtual video visits;
    - Ambulatory/portable infusion pumps that can be worn on the hip;
- Improved transitions of care
    - Ability to coach patients on their post-discharge care plan in the appropriate environment, with caregivers available, and with adequate time for teaching given provider to patient ratio

Caregivers:

Possible benefits may include helping others in the future by participating in this study.

Clinicians:

Possible benefits may include helping others in the future by participating in this study

Potential benefits to society

- New evidence-based care paradigm for acute care hospitalization in rural US.
- Lower total medical expenditure. Allows for redirection of resources to areas in need.
- Might eventually lead to reduction in total hospitals in the US.
- Provide needed randomized controlled data on the rural home hospital intervention to inform payment methods moving forward.

9.0 MONITORING AND QUALITY ASSURANCE

The BWH study team serves as this study's coordinating center. The study sites will be collecting the data and the BWH study team will be regularly monitoring data (not directly collecting data). There is a monitoring plan document attached to this study protocol which outlines in detail the monitoring steps that will be taken by the BWH study team and the study sites

Safety monitoring

If any of the following concerning safety events occur, a blinded monitoring committee will review the event to determine attribution to the intervention: fall with injury, medication error, DVT/PE, mortality during admission, transfer back to hospital (committee cannot be blinded for this endpoint).

If any of these events are felt to be due to the RHH intervention, they will be reported to the IRB with recommendations for appropriate actions to be taken. Decisions to modify the protocol or suspend the study will be made jointly by the study investigators, monitoring committee, and the IRB.

Outcomes monitoring

The RHH MD and RHH RN/paramedics will review quality and safety data daily as part of a rapid logistics improvement process.

Weekly, the RHH MD and RHH RN/paramedic will huddle to review quality and safety data as part of a rapid logistics improvement process.

Adverse event reporting guidelines

Please refer to the Monitoring Plan for detailed procedures

All unanticipated problems including adverse events denoted above under safety monitoring will be reported to the DSMB.

Safety data will be extracted periodically from the participant's medical records and will be retrieved every 6 months through the study; DSMB members will be reported in a semiannual summary safety report.

10.0 APENDIX

For the RHH RCT U.S. study sites we will be using the following definition of "rural", based on 2010 U.S. census data (15, 16):

- The Federal Office of Rural Health Policy (FORHP) at the Health Resources & Services Administration (HRSA) defines the following areas as "rural":
    - All non-metro counties (counties that are nonmetropolitan or micropolitan)
    - All metro census tracts with RUCA codes 4-10 and
    - Large area Metro census tracts of at least 400 sq. miles in area with population density of 35 or less per sq. mile with RUCA codes 2-3.

Health care providers who are in geographic areas that are defined as rural by the FORHP, are eligible to apply for, or receive services from, HRSA rural health grants.

For RHH RCT Canadian site (AHS), we will be using the following definition of "rural" based on the 2018 Alberta Health Services (AHS)/ Alberta Health (AH) official standard geographic areas. Rural includes "Large Rural Centres and Surrounding Areas" as defined by the AHS/AH (17):

- 10,000 to less than 25,000 population (Brooks, Canmore, Wetaskiwin, Camrose, Lloydminster, Cold Lake).

- All 5 areas have unique populations and industries but belong to the rural area.

11.0 REFERENCES

1. Hung WW, Ross JS, Farber J, Siu AL. Evaluation of the Mobile Acute Care of the Elderly (MACE) service. *JAMA Intern Med*. 2013;173(11):990-996. doi:10.1001/jamainternmed.2013.478.
2. Fong TG, Tulebaev SR, Inouye SK. Delirium in elderly adults: diagnosis, prevention and treatment. *Nat Rev Neurol*. 2009;5(4):210-220. doi:10.1038/nrneurol.2009.24.
3. 2014 National and State Healthcare-Associated Infections Progress Report.; 2016. http://www.cdc.gov/hai/surveillance/progress-report/index.html. Accessed April 19, 2016.
4. Counsell SR, Holder CM, Liebenauer LL, et al. Effects of a Multicomponent Intervention on Functional Outcomes and Process of Care in Hospitalized Older Patients: A Randomized Controlled Trial of Acute Care for Elders (ACE) in a Community Hospital. *J Am Geriatr Soc*. 2000;48(12):1572-1581. doi:10.1111/j.1532-5415.2000.tb03866.x.
5. Leff B, Burton L, Mader SL, et al. Hospital at home: feasibility and outcomes of a program to provide hospital-level care at home for acutely ill older patients. *Ann Intern Med*. 2005;143(11):798-808. http://www.ncbi.nlm.nih.gov/pubmed/16330791. Accessed February 15, 2016.
6. Cryer L, Shannon SB, Van Amsterdam M, Leff B. Costs for "hospital at home" patients were 19 percent lower, with equal or better outcomes compared to similar inpatients. *Health Aff (Millwood)*. 2012;31(6):1237-1243. doi:10.1377/hlthaff.2011.1132.
7. Levine DM, Ouchi K, Blanchfield B, Diamond K, Licurse A, Pu CT, and Schnipper JL. Hospital-Level Care at Home for Acutely Ill Adults: A Pilot Randomized Controlled Trial. *J Gen Intern Med*. 2018; 33(5):729-736. doi: 10.1007/s11606-018-4307-z

8 Levine DM, Ouchi K, Blanchfield B, Saenz A, Burke K, Paz M, et al. Hospital-Level Care at Home for Acutelly Ill Adults: A Randomized Controlled Trial. *Ann Intern Med* .2019. 21;172(2):77-85. doi: 10.7326/M19-0600

9 Bureau UC. What is Rural America?https://www.census.gov/library/stories/2017/08/rural-america.html. Published 2017. Accessed May 31, 2019.

10 Garcia MC, Rossen LM, Bastian B, et al. Potentially Excess Deaths from the Five Leading Causes of Death in Metropolitan and Nonmetropolitan Counties — United States, 2010–2017. MMWR Surveill Summ. 2019;68(10):1-11. doi:10.15585/mmwr.ss6810a1

11 Parker K, Horowitz J, Brown A, Fry R, Cohn D, Igielnik R. What Unites and Divides Urban, Suburban and Rural Communities.; 2018. https://www.pewsocialtrends.org/wpcontent/uploads/sites/3/2018/05/Pew-Research-Center-Community-Type-Full-Report-FINAL.pdf. Accessed May 31, 2019

12. Creditor MC. Hazards of hospitalization of the elderly. Ann Intern Med. 1993;118(3):219-223. http://www.ncbi.nlm.nih.gov/pubmed/8417639. Accessed May 31, 2019.
13. Joynt KE, Orav EJ, Jha AK. Mortality rates for Medicare beneficiaries admitted to critical access and non-critical access hospitals, 2002-2010. JAMA. 2013;309(13):1379-1387. doi:10.1001/jama.2013.2366

14 Joynt KE, Harris Y, Orav EJ, Jha AK. Quality of Care and Patient Outcomes in Critical Access Rural Hospitals. JAMA. 2011;306(1):45-52. doi:10.1001/jama.2011.902

15. Defining Rural Population | Official web site of the U.S. Health Resources & Services Administration [Internet]. [cited 2021 Nov 30]. Available from: https://www.hrsa.gov/rural-health/about-us/definition/index.html

16. Defining Rural Population | Official web site of the U.S. Health Resources & Services Administration. Accessed November 30, 2021. https://www.hrsa.gov/rural-health/about-us/definition/index.html

17. Alberta Health Services, Alberta Health. Official Standard Geographic Areas. January 2018. Accessed May 22, 2022. https://open.alberta.ca/dataset/a14b50c9-94b2-4024-8ee5-c13fb70abb4a/resource/70fd0f2c-5a7c-45a3-bdaa-e1b4f4c5d9a4/download/official-standard-geographic-area-document.pdf
