# Supplementary material for: Hospital-Level Care at Home for Adults Living in Rural Settings: A Randomized Clinical Trial
Source: JAMA Netw Open. 2025 Dec 1;8(12):e2545712. doi: 10.1001/jamanetworkopen.2025.45712 (PMC12670196; doi:10.1001/jamanetworkopen.2025.45712)
Supplement: Supplement 2. — eTable 1. Definition of Rural eTable 2a. Geographic Details by Site eTable 2b. Additional Geographic Details for Blessing Health System eTable 2c. Additional Geographic Details for Haward ARH Regional Medical Center eTable 2d. Additional Geographic Details for Wetaskiwin Hospital (Alberta Health Services) eTable 3. Population Density eTable 4. Detailed Inclusion and Exclusion Criteria eTable 5. Capabilities by Site eTable 6. Process Measures, Home Group eTable 7. Cost and Room and Board Details eTable 8. Patient Safety Measures eTable 9. Utilization Among Intervention Patients, by Home vs Hospital Phase of Care eTable 10. Baseline Patient Characteristics (Home in Less Than 3 Days of Brick-and-Mortar vs Control) eTable 11. Relative Cost of Home Hospital Care (Home in Less Than 3 Days of brick-and-mortar) to brick-and-Mortar Hospital Care eTable 12. Patient Health Care Use (Home in Less Than 3 Days of brick-and-mortar vs Control) eTable 13. Quality, Physical Activity, Functional Status, and Experience (Home in Less Than 3 Days of brick-and-mortar vs. Control) eAppendix 1. Detail Regarding Secondary Outcomes eAppendix 2. Table 1 Missing Data eAppendix 3. Table 4 Missing Data eReference [file jamanetwopen-e2545712-s002.pdf]

# Supplemental Online Content

Levine DM, Desai MP, Findeisen SM, et al. Hospital-level care at home for adults living in rural settings. *JAMA Netw Open.* 2025;8(12):e2545712.
doi:10.1001/jamanetworkopen.2025.45712

**eTable 1.** Definition of rural

**eTable 2** a. Geographic detals by site

**eTable 2** b. Additional geographic details for Blessing Health System

**eTable 2** c. Additional geographic details for Haward ARH Regional Medical Center

**eTable 2** d. Additional geographic details for Wetaskiwin Hospital (Alberta Health Services)

**eTable 3.** Population Density

**eTable 4.** Detailed inclusion and exclusion criteria

**eTable 5.** Capabilities by Site

**eTable 6.** Process Measures, Home Group

**eTable 7.** Cost and Room and Board Details

**eTable 8.** Patient Safety Measures

**eTable 9.** Utilization Among Intervention Patients, by Home vs Hospital Phase of Care

**eTable 10.** Baseline Patient Characteristics (Home in Less Than 3 Days of Brick-and-Mortar vs Control)

**eTable 11.** Relative cost of home hospital care (home in less than 3 days of brick-and-mortar) to brick-and-mortar hospital care

**eTable 12.** Patient health care use (home in less than 3 days of brick-and-mortar vs. control)a

**eTable 13.** Quality, physical activity, functional status, and experience (home in less than 3 days of brick-and-mortar vs. control)

**eAppendix 1.** Detail Regarding Secondary Outcomes

**eAppendix 2.** Table 1 Missing Data

**eAppendix 3.** Table 4 Missing Data

**eReference**

© 2025 Levine DM, et al. *JAMA Network Open*.

**eTable 1**. Definition of rural

| |
|---|
| For the Rural Home Hospital (RHH) Randomized Controlled Trial (RCT) United States study sites, the following definition of "rural" was used based on 2010 U.S. census data[1]:<br><br>• The Federal Office of Rural Health Policy (FORHP) at the Health Resources & Services Administration (HRSA) defines the following areas as "rural:"<br>  o All non-metro counties (counties that are nonmetropolitan or micropolitan)<br>  o All metro census tracts with RUCA codes 4-10 and<br>  o Large area Metro census tracts of at least 400 sq. miles in area with population density of 35 or less per sq. mile with RUCA codes 2-3.<br><br>Health care providers who are in geographic areas that are defined as rural by the FORHP, are eligible to apply for, or receive services from, HRSA rural health grants.<br><br>For RHH RCT Canadian site Alberta Health Services (AHS), the following definition of "rural" based on the 2018 AHS/ Alberta Health (AH) official standard geographic areas was used. Rural includes "Large Rural Centres and Surrounding Areas" as defined by the AHS/AH[2]:<br><br>• 10,000 to less than 25,000 population (Brooks, Canmore, Wetaskiwin, Camrose, Lloydminster, Cold Lake).<br>• All 5 areas have unique populations and industries but belong to the rural area. |

© 2025 Levine DM, et al. *JAMA Network Open*.

**eTable 2a**. Geographic details by site

| Characteristic | Blessing Health System[a] | Hazard Appalachian Regional Medical Center[a] (ARH) | Wetaskiwin Hospital (Alberta Health Services)[b, c] |
|---|---|---|---|
| Location | Quincy, Illinois, USA | Hazard, Kentucky, USA | Wetaskiwin City, Alberta, Canada |
| County where hospital is located | Adams County | Perry County | Wetaskiwin County |
| Other counties served/ Catchment area | See eTable 2b | See eTable 2c | See eTable 2d |
| County population | 63,767[3] | 27,367[3] | 12,594 (2021) |
| Population per square mile (2022) | 76.9[3] | 83.8[3] | 1,739 (2021)[4] |
| Median household income (2022) | $63,767[3] | $45,330[3] | $50,005 (2020)[4] |
| Persons in poverty (2022) | 13.4 %[3] | 27 %[3] | 12.8% (2020)[4] |
| Unemployment rate (2022) | 3.4%[5] | 5.3%[5] | 12.0% (2021)[4] |
| Primary care HPSA score[d] | 14[6] | 13[6] | Not applicable |
| RUCA[e] | 4,5,10[7] | 7,8,10[7] | Not applicable |
| CMS ambulance fee schedule code[f] | Rural[8] | Rural[8] | Not applicable |
| FAR[g] | 1[9] | 3[9] | Not applicable |
| HRSA eligibility for rural health funding | Eligible[10] | Eligible[10] | Not applicable |
| Percent of study patients living in county where hospital is located | 82.4% | 80.5% | 100% |
| Racial and ethnic groups | 0.6% AI/AN<br>1.3% Asian<br>5.7% Black<br>0.1% NHPI<br>2.1% two or more races[h]<br>94.7% White | 0.3% AI/AN<br>0.8% Asian<br>1% Black<br>0.2% NHPI<br>3.3% two or more races[h]<br>94.4% White | 18% Indigenous peoples<br>(12.8% First Nations<br>4.3% Métis<br>1.8% Inuit)<br>13.5% Other racialized population<br>(7.9% Filipino<br>1.5% South Asian<br>1.1% Black<br>3% some other racialized group)[i] |

[a]For US sites we considered multiple indices of rural, including the primary care HPSA score, RUCA, CMS ambulance fee schedule code, FAR and HRSA to help us determine if the hospital was rural or not.

[b]For RHH RCT Canadian site (AHS), we will be using the following definition of "rural" based on the 2018 Alberta Health Services (AHS)/ Alberta Health (AH) official standard geographic areas. Rural includes "Large Rural Centres and Surrounding Areas" as defined by the AHS/AH[11]

[c]Data in this column only reflects Wetaskiwin City, Alberta, Canada. See eTable 2d for geographic details of Wetaskiwin County No. 10, Alberta, Canada.

[d]HPSA designations are used to identify areas and population groups within the United States that are experiencing a shortage of health professionals. The primary factor used to determine a HPSA designation is the number of health professionals relative to the population with consideration of high need. Federal regulations stipulate that, in order to be considered as having a shortage of providers, an area must have a population-to-provider ratio of a certain threshold. Primary Care HPSAs can receive a score between 0-25 that considers the population to provider ratio, percent of population below the 100% federal poverty level, infant health index and travel time to nearest source of care (NSC). Scores closer to 0 indicate few needs and scores closer to 25 indicate greater needs as it is a shortage area.[6]

[e]Rural-Urban Commuting Area (RUCA) Codes: each census tract (or ZIP code) is assigned a primary code between 1-10 depending on metropolitan/micropolitan/small town/rural characteristics and basic commuting flows: 1 (Metropolitan area core: primary flow within an urbanized area (UA) - 10 (Rural areas: primary flow to a tract outside a UA or urban cluster)[7]

© 2025 Levine DM, et al. *JAMA Network Open*.

[f]Centers for Medicare and Medicaid Services (CMS) Ambulance Fee Schedule): The Medicare Part B Ambulance Fee Schedule (AFS) is a national fee schedule for ambulance services, base payment for ground ambulance transports increase when point-of-pickup zip code is designated as rural. CMS ambulance fee schedule codes are calculated from base rate and air mileage rate to determine if the area is "urban (lowest base rate and air mileage)", "rural", "super rural (highest base rate and air mileage)".
[g]FAR areas are defined in relation to the time it takes to travel by car to the edges of nearby Urban Areas (UAs). Four levels are necessary because rural areas experience degrees of remoteness at higher or lower population levels that affect access to different types of goods and services. Level one FAR codes coincide with a smaller number of people who find it hard to access "low order" goods and services, such as grocery stores, gas stations, and basic health-care services. Level four FAR codes indicate a higher degree of remoteness, based on access to goods and services.[9]
[h]From the 2023 American Community Survey 5-Year Estimates. Some proportions may not add up to 100% as they are estimates.[12]
[i]The census in Canada identifies number of indigenous people and "racialized groups" but it does not identify number of people who are White.[13]
Abbreviations: AI/AN = American Indian or Alaska Native; CMS = Centers for Medicare & Medicaid; FAR = frontier and remote area; HPSA = Health Professional Shortage Area; HRSA = Health Resources and Services Administration; NHPI = Native Hawaiian or Pacific Islander; RUCA = Rural-Urban Commuting Area; USA = United States of America

© 2025 Levine DM, et al. *JAMA Network Open*.

**eTable 2b**. Additional geographic details for Blessing Health System

| Characteristic | Blessing Health System | | | |
|---|---|---|---|---|
| Location | Quincy, Illinois, USA | | | |
| County | Pike County | Hancock County | Clark County (MO)[a] | Lewis County (MO)[a] |
| County population | 14,342[3] | 17,186[3] | 6,641[3] | 9,818[3] |
| Population per square mile (2022) | 17.7[3] | 22.2[3] | 13.1[3] | 19.9[3] |
| Median household income (2022) | \$55,514[3] | \$61,026[3] | \$53,362[3] | \$49,779[3] |
| Persons in poverty (2022) | 16.6%[3] | 12.3%[3] | 13.0%[3] | 13.6%[3] |
| Unemployment rate (2022) | 4.5%[5] | 4.1%[5] | 3.8%[5] | 2.9%[5] |
| Primary care HPSA score[b] | 10[6] | 15[6] | 14[6] | 18[6] |
| RUCA[c] | 7, 8, 9, 10[7] | 4, 5, 6, 7, 10[7] | 2, 5, 7, 8, 10[7] | 5, 10[7] |
| CMS ambulance fee schedule code[d] | Very rural[8] | Rural and very rural[8] | Very rural[8] | Rural and very rural[8] |
| FAR[e] | 1, 2, 3[9] | 1[9] | 1, 2, 3, 4[9] | 1, 2, 3, 4[9] |
| HRSA eligibility for rural health funding[8] | Eligible[10] | Eligible[10] | Eligible[10] | Eligible[10] |
| Percent of study patients at the site[f] | 0% | 0% | 5.9% | 9.8% |
| Racial and ethnic groups[g] | 0.3% Asian<br>0.5% Black<br>0.7% NHPI<br>0.6% Other<br>1.8% Two or more races<br>96.1% White | 0.1% AI/AN<br>0.3% Asian<br>0.8% Black<br>0.9% Other<br>2.8% Two or more races 95% White | 0.4% AI/AN<br>0.4% Asian<br>0.4% Black<br>0.2% Other<br>2.9% Two or more races<br>95.7% White | 0.1% AI/AN<br>0.5% Asian<br>3% Black<br>0.2% NHPI<br>4.0% Two or more races<br>92.2% White |

[a]These two counties are not in IL but are included in the catchment area of Blessing Health System.
[b]HPSA designations are used to identify areas and population groups within the United States that are experiencing a shortage of health professionals. The primary factor used to determine a HPSA designation is the number of health professionals relative to the population with consideration of high need. Federal regulations stipulate that, in order to be considered as having a shortage of providers, an area must have a population-to-provider ratio of a certain threshold. Primary Care HPSAs can receive a score between 0-25 that considers the population to provider ratio, percent of population below the 100% federal poverty level, infant health index and travel time to nearest source of care (NSC). Scores closer to 0 indicate few needs and scores closer to 25 indicate greater needs as it is a shortage area.[6]
[c]Rural-Urban Commuting Area (RUCA) Codes: each census tract (or ZIP code) is assigned a primary code between 1-10 depending on metropolitan/micropolitan/small town/rural characteristics and basic commuting flows: 1 (Metropolitan area core: primary flow within an urbanized area (UA) - 10 (Rural areas: primary flow to a tract outside a UA or urban cluster)[7]
[d]Centers for Medicare and Medicaid Services (CMS) Ambulance Fee Schedule): The Medicare Part B Ambulance Fee Schedule (AFS) is a national fee schedule for ambulance services, base payment for ground ambulance transports increase when point-of-pickup zip code is designated as rural. CMS ambulance fee schedule codes are calculated from base rate and air mileage rate to determine if the area is "urban (lowest base rate and air mileage)", "rural", "super rural (highest base rate and air mileage)".
[e]FAR areas are defined in relation to the time it takes to travel by car to the edges of nearby Urban Areas (UAs). Four levels are necessary because rural areas experience degrees of remoteness at higher or lower population levels that affect access to different types of goods and services. Level one FAR codes coincide with a smaller number of people who find it hard to access "low order" goods and services, such as grocery stores, gas stations, and basic health-care services. Level four FAR codes indicate a higher degree of remoteness, based on access to goods and services.[9]
[f]1 patient cared for was from Marion County.
[g]From the 2023 American Community Survey 5-Year Estimates. Some proportions may not add up to 100% as they are estimates.[12]
Abbreviations: AI/AN = American Indian or Alaska Native; CMS = Centers for Medicare & Medicaid; FAR = frontier and remote area; HPSA = Health Professional Shortage Area; HRSA = Health Resources and Services Administration; MO = Missouri; NHPI = Native Hawaiian or Pacific Islander; RUCA = Rural-Urban Commuting Area; USA = United States of America

© 2025 Levine DM, et al. *JAMA Network Open*.

**eTable 2c**. Additional geographic details for Hazard ARH Regional Medical Center

| **Characteristic** | **Hazard ARH Regional Medical Center** | | | |
|---|---|---|---|---|
| Location | Hazard, Kentucky, USA | | | |
| County | Knott county | Leslie county | Letcher county | Breathitt county |
| County population | 13,659[3] | 9,864[3] | 20,423[3] | 12,953[3] |
| Population per square mile (2022) | 40.5[3] | 26.2[3] | 63.8[3] | 27.9[3] |
| Median household income (2022) | $38,336[3] | $37,019[3] | $38,466[3] | $38,209[3] |
| Persons in poverty (2022) | 29.2%[3] | 30.1%[3] | 28.7%[3] | 30.1%[3] |
| Unemployment rate (2022) | 6.7%[5] | 6.9%[5] | 6.3%[5] | 7.5%[5] |
| Primary care HPSA score[a] | 16[6] | 20[6] | 19[6] | 16[6] |
| RUCA[b] | 10[7] | 10[7] | 5, 10[7] | 2, 7, 8, 10[7] |
| CMS ambulance fee schedule code[c] | Rural[8] | Rural[8] | Rural[8] | Rural and very rural[8] |
| FAR[d] | 1, 2, 3, 4[9] | 1, 2, 3, 4[9] | 1, 2, 3, 4[9] | 1, 2, 3, 4[9] |
| HRSA eligibility for rural health funding[8] | Eligible[10] | Eligible[10] | Eligible[10] | Eligible[10] |
| Percent of study patients at the site | 7.8% | 6.5% | 2.6% | 2.6% |
| Racial and ethnic groups[e] | 0.2% AI/AN<br>0.2% Asian<br>1.0% Black<br>0.6% Other<br>1.3% Two or more races<br>96.7% White | 0.3% Asian<br>0.5% Black<br>0.0% Other<br>2.2% Two or more races<br>96.9% White | 0.2% Asian<br>0.7% Black<br>0.3% Other<br>1.4% Two or more races<br>97.3% White | 0.5% AI/AN<br>1.1% Asian<br>0.6% Black<br>1.4% Other<br>98.5% White |

[a]HPSA designations are used to identify areas and population groups within the United States that are experiencing a shortage of health professionals. The primary factor used to determine a HPSA designation is the number of health professionals relative to the population with consideration of high need. Federal regulations stipulate that, in order to be considered as having a shortage of providers, an area must have a population-to-provider ratio of a certain threshold. Primary Care HPSAs can receive a score between 0-25 that considers the population to provider ratio, percent of population below the 100% federal poverty level, infant health index and travel time to nearest source of care (NSC). Scores closer to 0 indicate few needs and scores closer to 25 indicate greater needs as it is a shortage area.[6]

[b]Rural-Urban Commuting Area (RUCA) Codes: each census tract (or ZIP code) is assigned a primary code between 1-10 depending on metropolitan/micropolitan/small town/rural characteristics and basic commuting flows: 1 (Metropolitan area core: primary flow within an urbanized area (UA) - 10 (Rural areas: primary flow to a tract outside a UA or urban cluster)[7]

[c]Centers for Medicare and Medicaid Services (CMS) Ambulance Fee Schedule): The Medicare Part B Ambulance Fee Schedule (AFS) is a national fee schedule for ambulance services, base payment for ground ambulance transports increase when point-of-pickup zip code is designated as rural. CMS ambulance fee schedule codes are calculated from base rate and air mileage rate to determine if the area is "urban (lowest base rate and air mileage)", "rural", "super rural (highest base rate and air mileage)".

[d]FAR areas are defined in relation to the time it takes to travel by car to the edges of nearby Urban Areas (UAs). Four levels are necessary because rural areas experience degrees of remoteness at higher or lower population levels that affect access to different types of goods and services. Level one FAR codes coincide with a smaller number of people who find it hard to access "low order" goods and services, such as grocery stores, gas stations, and basic health-care services. Level four FAR codes indicate a higher degree of remoteness, based on access to goods and services.[9]

[e]From the 2023 American Community Survey 5-Year Estimates. Some proportions may not add up to 100% as they are estimates.[12]

Abbreviations: AI/AN = American Indian or Alaska Native; CMS = Centers for Medicare & Medicaid; FAR = frontier and remote area; HPSA = Health Professional Shortage Area; HRSA = Health Resources and Services Administration; RUCA = Rural-Urban Commuting Area

© 2025 Levine DM, et al. *JAMA Network Open*.

**eTable 2d.** Additional geographic details for Wetaskiwin Hospital (Alberta Health Services)

| **Characteristic** | **Wetaskiwin Hospital (Alberta Health Services)** |
|---|---|
| Location | Wetaskiwin, Alberta, Canada |
| County | Wetaskiwin County No. 10[a] |
| Population (2021) | 11,212[a] |
| Population per square mile (2021) | 9.3[a] |
| Median household income (2020) | $54,219[a] |
| Persons in poverty (2020) | 11.4%[a] |
| Unemployment rate (2021) | 9.9%[a] |
| Primary care HPSA score[b] | Not applicable |
| RUCA[c] | Not applicable |
| CMS ambulance fee schedule code[d] | Not applicable |
| FAR[e] | Not applicable |
| HRSA eligibility for rural health funding[8] | Not applicable |
| Percent of study patients at the site | 12.1% |
| Racial and ethnic groups[f] | 6.7% Indigenous peoples<br>(2.9% First Nations<br>3.6% Métis<br>0.1% Inuit)<br>2.2% Other visible racialized population<br>(0.6% Filipino<br>0.3% Latin American<br>0.3% Arab<br>1.7% some other racialized group) |

[a]Geographic details only include Wetaskiwin County No. 10 and not Wetaskiwin City or Indigenous people's reservation data as noted by Statistics Canada.[1] See eTable 2 for details about Wetaskiwin City.

[b]HPSA designations are used to identify areas and population groups within the United States that are experiencing a shortage of health professionals. The primary factor used to determine a HPSA designation is the number of health professionals relative to the population with consideration of high need. Federal regulations stipulate that, in order to be considered as having a shortage of providers, an area must have a population-to-provider ratio of a certain threshold. Primary Care HPSAs can receive a score between 0-25 that considers the population to provider ratio, percent of population below the 100% federal poverty level, infant health index and travel time to nearest source of care (NSC). Scores closer to 0 indicate few needs and scores closer to 25 indicate greater needs as it is a shortage area.[2]

[c]Rural-Urban Commuting Area (RUCA) Codes: each census tract (or ZIP code) is assigned a primary code between 1-10 depending on metropolitan/micropolitan/small town/rural characteristics and basic commuting flows: 1 (Metropolitan area core: primary flow within an urbanized area (UA) - 10 (Rural areas: primary flow to a tract outside a UA or urban cluster)[3]

[d]Centers for Medicare and Medicaid Services (CMS) Ambulance Fee Schedule): The Medicare Part B Ambulance Fee Schedule (AFS) is a national fee schedule for ambulance services, base payment for ground ambulance transports increase when point-of-pickup zip code is designated as rural. CMS ambulance fee schedule codes are calculated from base rate and air mileage rate to determine if the area is "urban (lowest base rate and air mileage)", "rural", "super rural (highest base rate and air mileage)".

[e]FAR areas are defined in relation to the time it takes to travel by car to the edges of nearby Urban Areas (UAs). Four levels are necessary because rural areas experience degrees of remoteness at higher or lower population levels that affect access to different types of goods and services. Level one FAR codes coincide with a smaller number of people who find it hard to access "low order" goods and services, such as grocery stores, gas stations, and basic health-care services. Level four FAR codes indicate a higher degree of remoteness, based on access to goods and services.[9]

[f]The census in Canada identifies number of indigenous people and "racialized groups"but does not identify number of people who are White.[4]

Abbreviations: CMS = Centers for Medicare & Medicaid; FAR = frontier and remote area; HPSA = Health Professional Shortage Area; HRSA = Health Resources and Services Administration; No. = number; RUCA = Rural-Urban Commuting Area

© 2025 Levine DM, et al. *JAMA Network Open*.

**eTable 3**. Population Density

| Population Density (people per sq. mile)[a] | Home (n=79) | Control (n = 82) |
|---|---|---|
| 0-24 | 23 | 21 |
| 25-49 | 10 | 8 |
| 50-74 | 11 | 3 |
| 75-99 | 2 | 5 |
| 100-124 | 15 | 22 |
| 125-149 | 10 | 7 |
| 150+ | 8 | 16 |

[a]Based on the zip codes of each participant, the county that they were in was identified. Based on the county, we identified the population density per sq. mile from the US Census for US sites and Statistics Canada for the Canadian site.[5,11]Abbreviations: sq = square

© 2025 Levine DM, et al. *JAMA Network Open*.

**eTable 4**. Detailed inclusion and exclusion criteria

**Patient inclusion criteria:**

**Clinical**

- >=18 years old.
- Primary or possible diagnosis of any infectious process, heart failure exacerbation, asthma and chronic obstructive pulmonary disease exacerbation, atrial fibrillation with rapid ventricular response, diabetes and its complications, venous thromboembolism (therapeutic anticoagulation and concomitant monitoring), gout exacerbation, chronic kidney disease with volume overload, hypertensive urgency, end of life / desires only medical management.

**Environmental**

- Lives in a rural area that can be served by the RHH team.
- Has capacity to consent to study OR can assent to study and has proxy who can consent (see subject enrollment, below).
- Can identify a potential caregiver who agrees to stay with patient for first 24 hours of admission. Caregiver must be competent to call care team if a problem is evident to her/him. After 24 hours, this caregiver should be available for as-needed spot checks on the patient.
    - This criterion may be waived for highly competent patients at the patient and clinician's discretion.

**Patient exclusion criteria:**

**Clinical**

- Acute delirium, as determined by the Confusion Assessment Method.
- Cannot establish peripheral access by any means.
- Secondary condition: active non-melanoma/prostate cancer, end-stage renal disease, acute myocardial infarction, acute cerebral vascular accident, acute hemorrhage (unless part of end of life pathway).
- Primary diagnosis requires multiple or routine administrations of intravenous narcotics for pain control.
- Cannot independently ambulate to bedside commode, unless home-based aides are available.
- As deemed by on-call MD, patient likely to require any of the following procedures that have not already occurred: computed tomography, magnetic resonance imaging, endoscopic procedure, blood transfusion, cardiac stress test, or surgery (unless these can be coordinated with appropriate facilities during the home hospitalization).
- For pneumonia:
    - Most recent CURB65 > 3: new confusion, BUN > 19mg/dL, respiratory rate>=30/min, systolic blood pressure<90mmHg, Age>=65 (<14% 30-day mortality)
    - Most recent SMRTCO > 2: systolic blood pressure < 90mmHg (2pts), multilobar CXR involvement (1pt), respiratory rate >= 30/min, heart rate >= 125, new confusion, oxygen saturation <= 90% (<10% chance of intensive respiratory or vasopressor support)[16]
    - Absence of clear infiltrate on imaging
    - Cavitary lesion on imaging
    - Pulmonary effusion of unknown etiology
    - $O_2$ saturation < 90% despite 5L $O_2$
- For heart failure:
    - Has a left ventricular assist device
    - GWTG-HF[17] (>10% in-hospital mortality) or ADHERE[18] (high risk or intermediate risk 1)*
    - Severe pulmonary hypertension
- For complicated urinary tract infection:
    - Absence of pyuria
    - Most recent qSOFA > 1 (SBP≤100 mmHg, RR≥22, GCS<15 [any AMS]) (if sepsis, >10% mortality)[19]
- For other infection
    - Most recent qSOFA > 1 (SBP≤100 mmHg, RR≥22, GCS<15 [any AMS]) (if sepsis, >10% mortality)[19]
- For COPD
    - BAP-65 score > 3 (BUN>25, altered mental status, HR>109, age>65) (<13% chance in-hospital mortality): exercise caution

© 2025 Levine DM, et al. *JAMA Network Open*.

- For asthma
    - Peak expiratory flow < 50% of normal: exercise caution
- For diabetes and its complications
    - Requires IV insulin
- For hypertensive urgency
    - Systolic blood pressure > 190 mmHg
    - Evidence of end-organ damage; for example, acute kidney injury, focal neurologic deficits, myocardial infarction
- For atrial fibrillation with rapid ventricular response
    - Likely to require cardioversion
    - New atrial fibrillation with rapid ventricular response
    - Unstable blood pressure, respiratory rate, or oxygenation
    - Despite IV beta and/or calcium channel blockade in the emergency department, HR remains > 125 and SBP remains different than baseline
    - Less than 1 hour of time has elapsed with HR < 125 and SBP similar or higher than baseline
- Home hospital census is full

*GWTG-HF: AHA Get with the Guidelines: SBP, BUN, Na, Age, HR, Black race, COPD

ADHERE: Acute decompensated heart failure national registry: BUN, creatinine, SBP

**Environmental**

- Undomiciled
- No working heat (October-April), no working air conditioning if forecast > 80°F (June-September), or no running water
- On methadone requiring daily pickup of medication
- In police custody
- Resides in facility that provides on-site medical care (e.g., skilled nursing facility)
- Domestic violence screen positive[8]

Abbreviations: ADHERE = Acute Decompensated Heart Failure National REgistry; BUN = blood urea nitrogen; COPD = Chronic Obstructive Pulmonary Disease; CURB-65 = confusion, uremia, respiratory rate, BP, age ≥ 65 years; CXR = chest x-ray; GWTG-HF = Get With The Guidelines®-Heart Failure; HR = heart rate; qSOFA = Quick Sequential Organ Failure Assessment; Na = sodium; RHH = rural home hospital; SBP = Spontaneous bacterial peritonitis; SMRTCO = systolic blood pressure, multilobar chest radiography involvement, respiratory rate, tachycardia, confusion, and oxygenation;

© 2025 Levine DM, et al. *JAMA Network Open*.

**eTable 5**. Capabilities by site

| Capability | Blessing | ARH | AHS |
|---|---|---|---|
| Admission hours | 9am-5pm, Monday-Friday | 9am-4pm, 7d/wk | Home between 7am-9pm, Monday-Friday |
| Inputs | ED, inpatient ward | ED, inpatient ward | ED, inpatient ward |
| Transportation to home | BLS, personal car | ARH wheelchair van, personal car | BLS, personal car |
| Catchment | 60-minute radius by road | 30-minute radius or 20 miles by road | 60-minute radius by road |
| Video communication | Yes | Yes | Yes |
| Internet connectivity[a] | 4G-enabled tablet | 4G-enabled tablet | 4G-enabled tablet |
| In-home team[b] | Acute-care RN twice daily | Acute-care RN twice daily | Community paramedic[c] / RN twice daily (once in-home, once remote) |
| Remote team[d] | Hospitalist once daily | General internist or hospitalist APP once daily | Hospitalist once daily |
| On-demand medications | In-home lock box | In-home lock box | Formulary carried by community paramedic |
| Hospital medication origin | Inpatient pharmacy | Inpatient pharmacy | Inpatient and community pharmacy |
| Urgent after-hours response | Remote response, then back to hospital via 911 (possible MD to home) | Remote response, then back to hospital via 911 | Remote response, then back to hospital via 911 |
| Emergent response | 911 | 911 | 911 |
| Biometric monitoring | Continuous (Biofourmis) | Continuous (Biofourmis) | Intermittent (no telemetry) (Cloud Dx) |
| IV infusions | Yes | Yes | Yes |
| Supplemental oxygen therapy | Yes | Yes | Yes |
| Nebulizer therapy | Yes | Yes | Yes |
| Imaging | Hospital round trip | Hospital round trip | Hospital round trip |
| Laboratory | ISTAT + core laboratory | n | ISTAT + core laboratory |
| Advanced vascular access placement | Hospital round trip | Hospital round trip | Hospital round trip |
| Advanced vascular access | Yes | Yes | Yes |
| Physical, occupational, speech therapy | In-home | Remote offering, with round trip to hospital for more extensive treatment | In-home |
| 12-lead ECG | Hospital round trip | In-home | In-home |
| Urinary catheter | In-home | In-home | In-home |
| Specialty consultation | Virtual consultation depending on consultation | Virtual consultation depending on consultation | Virtual consultation depending on consultation |

[a]Sites provided multi-carrier 4G connectivity to the patient's home.
[b]Facilitated the physician assessment and interaction and performed point-of-care blood diagnostics, IV administration, and respiratory therapies.
[c]Community paramedics are mobile integrated health paramedics who are specially trained paramedics with a broader scope of practice than traditional paramedics.
[d]If needed, patients could receive additional visits. The team was available 24/7 for any urgent issues.
Abbreviations: AHS = Alberta Health Services; APP = advanced practice provider; ARH = Appalachian Regional Healthcare; BLS = basic life support; d/wk = days per week; ECG = electrocardiogram; ED = emergency department; ICU = intensive care unit;  = in vitro whole-blood analyzer; IV = intravenous; MD = Doctor of Medicine; RN = registered nurse; 4G = fourth-generation wireless

© 2025 Levine DM, et al. *JAMA Network Open*.

© 2025 Levine DM, et al. *JAMA Network Open*.

**eTable 6**. Process measures, home group

| Measure | Home (n=79)[a] |
|---|---|
| Number of RN/CP visits per day, n, mean (SD)[b] | 1.62 (0.23) |
| Number of RN/CP visits per day, virtual, n (SD)[b] | 0.10 (0.22) |
| Number of RN/CP visits per day, in-person, n (SD)[b] | 1.52 (0.35) |
| RN/CP travel time per day, min (SD)[c] | 52.72 (28.75) |
| Duration of admission RN/CP visit, min (SD)[d] | 78.25 (41.35) |
| Duration of subsequent RN/CP visit, in-person, min (SD)[d] | 61.67 (23.37) |
| Duration of subsequent RN/CP visit, virtual, min (SD)[e] | 34.67 (43.09) |
| Insufficient handoff, n (%)[b] | - |
| Documentation error, n (%)[b] | - |
| Equipment malfunction, n (%)[b] | 19 (25.7) |
| Number of "on call" MD interactions per day, n (SD)[c] | 0.04 (0.13) |

Abbreviations: CP = community paramedic; MD = Doctor of Medicine; RN = registered nurse; SD = standard deviation
[a]Only includes home patients as most of the process measures are only applicable to those participants.
[b]For 5 patients, these data are either missing or the patient did not receive the intervention.
[c]For 7 patients, these data are either missing or the patient did not receive the intervention.
[d]For 6 patients, these data are either missing or the patient did not receive the intervention.
[e]For 64 patients, these data are missing because the patient did not receive the intervention or have a subsequent RN/CP visit.

© 2025 Levine DM, et al. *JAMA Network Open*.

**eTable 7**. Cost and room and board details[a,b]

| | Site | | | | | | | | |
|---|---|---|---|---|---|---|---|---|---|
| Cost Category[c] | Blessing | | | ARH | | | AHS[d] | | |
| | Intervention | Control | | Intervention | Control | | Intervention | Control | |
| ***Labor*** | Cost | R&B | Not R&B | Cost | R&B | Not R&B | Cost | R&B | Not R&B |
| Nurse | x | x | | x | x | | x | x | |
| Aide | N/A | x | | N/A | x | | | x | |
| Occupational therapist[e] | x | | x | x | | x | x | x | |
| Physical therapist[e] | x | | x | x | | x | x | x | |
| Social worker | N/A | x | | x | x | | N/A | N/A | |
| Nurse-level case management/care coordination | x[f] | x | | x[f] | x | | x[f] | x | |
| Medical record maintenance | EST | x | | EST | x | | EST | x | |
| Housekeeping services | N/A | x | | N/A | x | | N/A | x | |
| Trash and biohazard disposal | N/A | x | | N/A | x | | N/A | x | |
| ***Supplies*** | | | | | | | | | |
| Utilities (electricity, water, television, phone, etc.) | N/A | x | | N/A | x | | N/A | x | |
| Routine supplies[g] | x | x | | x | x | | x | x | |
| Wound care[g] | EST | x | EST | EST | x | x | EST | x | |
| Dressings[g] | EST | x | EST | EST | x | x | EST | x | |
| IV care | EST | x | | EST | x | | EST | x | |
| Surgical supplies for fractures and dislocations (splints, casts, not take-home supplies) | N/A | | x | N/A | | x | N/A | x | |
| Disposable supplies not considered routine | x | | x | x | | x | x | x | |
| Prosthetics | x | | x | x | | x | x | x | |
| Supplies for diagnostic tests | x | | x | x | | x | x | x | |
| Oxygen | x | | x | x | | x | EST | x | |
| Nebulizer | x | | x | x | | x | x | x | |
| Monitoring equipment | x | x | | x | x | | x | x | |
| Communication equipment | x | x | | x | x | | x | x | |
| Food and PO dietary requirements | x | x | | x | x | | x | x | |
| Other | x | | x | x | | x | x | | x |

© 2025 Levine DM, et al. *JAMA Network Open*.

| | | | | | | | | | |
|---|---|---|---|---|---|---|---|---|---|
| ***Medications*** | x | | x | x | | x | EST | | EST |
| ***Diagnostics*** | | | | | | | | | |
| Imaging | | | | | | | | | |
| Facility-based | x | | x | x | | x | EST | | EST |
| Point-of-care | x | | x | x | | x | EST | | EST |
| Laboratory tests | | | | | | | | | |
| Facility-based | x | | x | x | | x | x | | x |
| Point-of-care | x | | x | x | | x[h] | x | | x |
| ***Transport*** | | | | | | | | | |
| Patient | x | n/a | n/a | x | n/a | n/a | x | n/a | n/a |
| RN | x | n/a | n/a | x | n/a | n/a | x | n/a | n/a |
| Parking | x | n/a | n/a | x | n/a | n/a | x | n/a | n/a |

Abbreviations: AHS = Alberta Health Services; ARH =Appalachian Regional Healthcare; EST= estimate; IV = intravenous; N/A = not applicable; R&B = room and board; RN = registered nurse.

[a]Identifies if the supply is included in the room and board charge or if the cost is billed separately.

[b]One site sent resource intensity weights, which was used alongside direct costs (not covered in the RIW), to determine the overall cost of admission

[c]EST indicates that we had to estimate the cost on a per day basis. X indicates that we had the exact cost.

[d]For costs in 2022, the conversion rate of 0.76 to 1 (CAD to USD)[14] was used and for costs in 2023, the conversion rate of 0.74 to 1 (CAD to USD)[15] was used.

[e]Some of these costs were estimated for the intervention patients.

[f]Included in the nurse cost, not a separate cost

[g]Depending on a patient's wound requirements, the routine, wound and dressing supplies may be part of the room and board cost or they may be charged and estimated separately.

[h]Glucose tests were not included in the cost data and had to be added back. The glucose estimate was $8.09 USD per test.

© 2025 Levine DM, et al. *JAMA Network Open*.

**eTable 8**. Patient safety measures

| Measure | Home (n = 79) | Control (n = 82) |
|---|---|---|
| Fall, n (%)[a] | 2 (2.56) | 1 (1.23) |
| Delirium, n (%)[a] | - | - |
| DVT/PE, n (%)[a] | - | - |
| New pressure ulcer, n (%)[a] | 1 (1.28) | - |
| Thrombophlebitis at peripheral IV site, n (%)[a] | 1 (1.28) | 1 (1.23) |
| New arrhythmia, n (%)[a] | 3 (3.85) | - |
| Hypokalemia, n (%)[a] | 3 (3.85) | 4 (4.94) |
| Acute kidney injury, n (%)[a] | - | - |
| Medication error, n (%)[a] | 1 (1.28) | 4 (4.94) |
| Loss of consciousness, n (%)[a] | - | - |
| Escalation to ICU, n (%) | - | - |
| Death (unplanned) during admission, n (%) | - | - |
| Death (unplanned) 30 days after admission, n (%) | - | - |
| Death (all-cause) during admission, n (%) | - | - |
| Death (all-cause) 30 days after admission, n (%) | - | - |

Abbreviations: DVT = deep vein thrombosis; PE = pulmonary embolism; IV = intravenous; ICU = intensive care unit
Note: Safety measures in the home group include time in both BAM and home.
[a]For 1 intervention and 1 control patient, these data are missing.

© 2025 Levine DM, et al. *JAMA Network Open*.

**eTable 9**. Utilization among intervention patients, by home vs hospital phase of care[a]

| Measure[b] | Utilization while in the BAM hospital[c] (n = 74) | Utilization while at home[d] (n = 74) | p-value |
|---|---|---|---|
| *During acute care episode* | | | |
| Length of stay, mean (SD) | 4.28 (4.37) | 2.61 (1.69) | 0.001 |
| Intravenous medication, n (%) | 71 (95.9) | 60 (81.1) | 0.01 |
| Imaging, n (%) | 52 (70.3) | 15 (20.3) | <0.001 |
| Oxygen requirement, n (%) | 39 (52.7) | 21 (28.4) | <0.001 |
| Nebulizer requirement, n (%) | 34 (45.9) | 23 (31.1) | <0.001 |
| Lab orders per day, mean (SD) | 7.59 (3.67) | 1.81 (1.95) | <0.001 |
| Consultant sessions, n (%) | 31 (41.9) | 5 (6.8) | <0.001 |
| Physical or occupational therapy, n (%) | 17 (23.0) | 1 (1.4) | 0.004 |
| *Safety events* | | | |
| Fall, n (%) | 1 (1.4) | 2 (2.7) | 0.35 |
| Delirium, n (%) | - | - | |
| DVT/PE, n (%) | - | - | |
| New pressure ulcer, n (%) | - | 1 (1.4) | 0.94 |
| Thrombophlebitis at peripheral IV site, n (%) | - | - | |
| New arrhythmia, n (%) | 2 (2.7) | 1 (1.4) | 0.58 |
| Hypokalemia, n (%) | 1 (1.4) | 2 (2.7) | 0.58 |
| Acute kidney injury, n (%) | - | - | |
| Medication error, n (%) | 1 (1.4) | - | 0.94 |
| Unanticipated mortality, n (%) | - | - | |
| Loss of consciousness, n (%) | - | - | |
| Escalation to ICU, n (%) | - | - | |
| Death (unplanned) during admission, n (%) | - | - | |
| Death (unplanned) 30 days after admission, n (%) | - | - | |
| Death (all-cause) during admission, n (%) | - | - | |
| Death (all-cause) 30 days after admission, n (%) | - | - | |
| *Quality and safety of care* | | | |
| Any safety event, n (%) | 5 (6.8) | 6 (8.1) | 0.74 |
| Pain score, mean (SD)[e, f] | 2.53 (2.46) | 1.35 (2.09) | <0.001 |
| Inappropriate medication use, n (%)[g] | 29 (39.2) | 14 (18.9) | <0.001 |
| Urinary catheter use, n (%) | 2 (2.7) | 3 (4.1) | 0.67 |
| Restraint use, n (%) | 1 (1.4) | - | 0.94 |
| *Cost (percent change %, p-value)* | | | |
| Acute care episode | | | |
| Unadjusted cost (95% CI) | - | -56.45 (-66.75, -42.96) | <0.001 |
| Adjusted mean cost (95% CI)[h] | - | -55.28 (-66.1, -41) | <0.001 |
| Unadjusted cost per day (95% CI) | - | -47.89 (-58.1, -35.18) | <0.001 |
| Adjusted mean cost per day (95% CI)[i] | - | -49.38 (-60.61, -34.95) | <0.001 |

[a]For patients who were randomized to the intervention, healthcare use was broken down into whether the patient used it while admitted to the traditional brick and mortar (hospital phase) or whether the patient used it while at home (home phase).
[b]Values are numbers (percentages) unless otherwise indicated.
[c]Indicates if the patient received the care while they were in the traditional brick and mortar hospital.
[d]Indicates if the patient received the care while they were receiving hospital level care at home.
[e]Range, 0 –10 where higher scores indicate greater pain.
[f]For one patient in BAM and ten patients at home, this data is missing.
[g]Using the updated Beers Criteria (29).
[h]Adjusted by a fixed effect, which accounts for difference across the three sites.
Abbreviations: BAM = brick and mortar; DVT = deep vein thrombosis; PE = pulmonary embolism; ICU = intensive care unit; SD = standard deviation; CI = confidence interval

© 2025 Levine DM, et al. *JAMA Network Open*.

**eTable 10.** Baseline Patient Characteristics (Home in Less Than 3 Days of Brick-and-Mortar vs Control))[a]

| Characteristic | Home <3d BAM (n=40) | Control (n=82) | p-value |
|---|---|---|---|
| Age, mean (SD) yrs. | 64.7 (16.21) | 64.94 (14.11) | 0.94 |
| Sex | | | |
| Female | 23 (57.5) | 50 (60.98) | 0.72 |
| Male | 17 (42.5) | 32 (39.02) | |
| Race/ethnicity | | | |
| Asian | 1 (2.5) | - | 0.99 |
| Black | 1 (2.5) | 2 (2.44) | |
| Other[b] | - | 4 (4.88) | |
| White | 38 (95) | 76 (92.68) | |
| Partner status | | | |
| Single | 5 (12.5) | 11 (13.41) | 0.88 |
| Married/life-partner | 21 (52.5) | 43 (52.44) | |
| Divorced | 8 (20) | 11 (13.41) | |
| Widowed | 5 (12.5) | 15 (18.29) | |
| Other | 1 (2.5) | 2 (2.44) | |
| Lives alone[c] | 13 (33.33) | 22 (27.16) | 0.49 |
| Had home health aide[c] | 1 (2.56) | 2 (2.47) | 0.98 |
| Primary language | | | |
| English | 40 (100) | 82 (100) | |
| Other | | | |
| Health insurance[d] | | | |
| Private | 2 (5) | 6 (7.32) | 0.93 |
| Medicare | 25 (62.5) | 48 (58.54) | |
| Medicaid | 9 (22.5) | 15 (18.29) | |
| Medicare and Medicaid | 4 (10) | 12 (14.63) | |
| None | - | 1 (1.22) | |
| Education[e] | | | |
| Less than high school | 7 (17.95) | 17 (20.73) | 0.50 |
| High school | 17 (43.59) | 37 (45.12) | |
| Completed some college | 6 (15.38) | 18 (21.95) | |
| Associates degree | 6 (15.38) | 3 (3.66) | |
| Bachelor’s degree (4-year) | 3 (7.69) | 5 (6.1) | |
| Master’s degree or higher | - | 2 (2.44) | |
| Employment | | | |
| Employed | 8 (20) | 14 (17.07) | 0.73 |
| Unemployed | 8 (20) | 11 (13.41) | |
| Retired | 20 (50) | 45 (54.88) | |
| Other | 4 (10) | 12 (14.63) | |
| Smoking status | | | |
| Yes | 16 (40) | 28 (34.15) | 0.39 |
| No | 22 (55) | 53 (64.63) | |
| Don’t know | 2 (5) | 1 (1.22) | |
| Diagnosis | | | |
| Pneumonia | 8 (20) | 15 (18.29) | 0.97 |
| Cellulitis | 5 (12.5) | 11 (13.41) | |
| Complicated urinary tract infection/pyelonephritis | 2 (5) | 8 (9.76) | |
| Other infection | 5 (12.5) | 17 (20.73) | |
| Heart failure | 7 (17.5) | 12 (14.63) | |
| Asthma | 4 (10) | 0 (0) | |

© 2025 Levine DM, et al. *JAMA Network Open*.

| | | | |
|---|---|---|---|
| Chronic obstructive pulmonary disease | 8 (20) | 18 (21.95) | |
| Other[f] | 1 (2.5) | 1 (1.22) | |
| Mean comorbid condition count (SD)[c, g], admission | 3.97 (2.21) | 3.95 (2.08) | 0.96 |
| Mean PRISMA frailty score (SD)[h, i], admission | 2.63 (1.44) | 2.68 (1.61) | 0.87 |
| Mean 8-Item Interview to Differentiate Aging and Dementia score (SD)[j, k], admission | 1.5 (2.05) | 1.39 (2.21) | 0.80 |
| Mean PHQ-2 score (SD)[c, l], admission | 1.41 (1.62) | 1.3 (1.59) | 0.71 |
| Mean PROMIS emotional support score (SD)[c, m], admission | 57.53 (9.03) | 58.66 (7.01) | 0.49 |
| Mean Brief Health Literacy Screening Tool score (SD)[n, o], admission | 15.54 (3.73) | 16.05 (4.23) | 0.50 |
| Mean EuroQol VAS score (SD)[c, p], admission | 57.26 (23.39) | 56.63 (20.01) | 0.89 |
| Mean ADLs on admission (SD)[n, q] | 5.77 (0.74) | 5.6 (1.25) | 0.36 |
| Mean IADLs on admission (SD)[r, s] | 6.67 (2.06) | 6.64 (2.06) | 0.94 |
| Full code status (yes) | 34 (89.47) | 77 (95.06) | 0.28 |
| Mean outpatient medications (SD)[t], n | 7.95 (4.96) | 7.25 (6.45) | 0.52 |
| Mean readmission risk score on discharge (SD)[u] | 3.03 (1.98) | 3.39 (1.92) | 0.34 |
| Admitted to hospital in past 6 month | 18 (45) | 33 (40.24) | 0.62 |
| Visited ED in past 6 months | 28 (70) | 50 (60.98) | 0.34 |

[a]Values are numbers (percentages) unless otherwise indicated. Percentages may not sum to 100 due to rounding.
[b]Other includes those who identified as Native People and Middle Eastern.
[c]For 1 intervention and 1 control patient, these data are missing.
[d]Medicare counts include Canadian provincial insurance.
[e]For 1 intervention patient, this data is missing.
[f]Includes a viral illness and unknown diagnosis.
[g]Count of the patient's chronic comorbid conditions, out of the 20 conditions considered chronic by the Health and Human Services Office of the Assistant Secretary of Health (31).
[h]For 2 intervention and 1 control patient, these data are missing.
[i]Range, 0 –7, where scores >2 indicate frailty.
[j]Range, 0 – 8, where scores >1 indicate cognitive impairment.
[k]For 2 intervention and 3 control patient, these data are missing.
[l]Range, 0 – 6, where scores >2 indicate depression.
[m]Range, 25.7 –62, where scores >50.8 indicate better-than-average emotional support.
[n]For 1 intervention and 2 control patients, these data are missing.
[o]Range, 4 –20, where scores of 4 –12 indicate limited health literacy, scores of 13–16 indicate marginal health literacy, and scores of 17–20 indicate adequate health literacy.
[p]Range, 0 –100, where higher scores indicate better health.
[q]Range, 0 – 6, where higher scores indicate more independence.
[r]For 1 intervention and 5 control patients, these data are missing.
[s]Range, 0 – 8, where higher scores indicate more independence.
[t]For 6 control patients and 2 intervention patients, these data are missing.
[u]Range, 0 – 13, where scores of 0 – 4 indicate low risk of 30-day readmission, scores of 5 – 6 indicate intermediate risk of 30-day readmission, and scores greater than 7 indicate a high risk of 30-day readmission
Abbreviations: ADL = activity of daily living; BAM = brick-and-mortar; ED = emergency department; IADL = instrumental ADL; PHQ-2 = Patient Health Questionnaire 2; PRISMA = Program of Research to Integrate the Services for the Maintenance of Autonomy; PROMIS = Patient-Reported Outcomes Measurement Information System; SD = standard deviation; VAS = visual analogue scale; yrs = years

© 2025 Levine DM, et al. *JAMA Network Open*.

**eTable 11**. Relative cost of home hospital care (home in less than 3 days of brick-and-mortar) to brick-and-mortar hospital care

| Cost (95% CI) | Percent change[a] | p-value |
|---|---|---|
| Acute care episode | | |
| Unadjusted cost (95% CI) | -26.99 (-44.55, -3.88) | 0.03 |
| Adjusted mean cost (95% CI)[b] | -20.68 (-38.36, 2.06) | 0.08 |
| 30 d after acute care episode | | |
| Unadjusted cost (95% CI) | -33.53 (-66.53, 32.01) | 0.24 |
| Adjusted mean cost (95% CI)[b] | -30.47 (-68.57, 53.81) | 0.38 |
| Acute care episode and 30 d after acute care episode | | |
| Unadjusted cost (95% CI) | -27.98 (-46.57, -2.92) | 0.03 |
| Adjusted mean cost (95% CI)[b] | -27.06 (-44.80, -3.61) | 0.03 |

[a]Calculated as [(ratio of mean home to mean hospital – 1) × 100]. If percentage of change is negative, home group costs less; if percentage of change is positive, control group costs less.
[b]Adjusted for site as a fixed effect.
Abbreviations: CI = confidence interval

© 2025 Levine DM, et al. *JAMA Network Open*.

**eTable 12**. Patient health care use (home in less than 3 days of brick-and-mortar vs. control)[a]

| Measure | Home <3d BAM (n=40) | Control (n = 82) | p-value |
|---|---|---|---|
| *During acute care episode[b]* | | | |
| Length of stay (LOS), mean (SD) | 3.65 (1.75) | 5.38 (4.36) | 0.002 |
| LOS in BAM, mean (SD)[c] | 1.43 (0.55) | - | 0.34 |
| LOS at home, mean (SD)[d] | 2.23 (1.78) | - | 0.34 |
| Intravenous medication, n (%)[e] | 40 (100) | 77 (95.06) | 0.81 |
| Imaging, n (%)[f] | 19 (47.5) | 61 (74.39) | 0.01 |
| Oxygen requirement, n (%) | 18 (45) | 48 (58.54) | 0.17 |
| Nebulizer requirement, n (%)[e] | 17 (42.5) | 47 (58.02) | 0.12 |
| Lab orders per day, mean (SD) | 6.05 (3.82) | 9.16 (4.84) | <0.001 |
| Consultant session, n (%) | 9 (22.5) | 49 (59.76) | <0.001 |
| Physical or occupational therapy, n (%) | 5 (12.5) | 20 (24.39) | 0.14 |
| Disposition, n (%)[e] | | | |
| Routine | 34 (85) | 66 (81.48) | 0.96 |
| Home health | 6 (15) | 8 (9.88) | |
| Home hospice | - | 2 (2.47) | |
| Other | - | 5 (6.17) | |
| *30 days after acute care episode* | | | |
| 30-d Days at home, mean (SD) | 29.15 (2.59) | 28.43 (3.43) | 0.19 |
| 30-d Unplanned readmission, n (%) | 3 (7.5) | 14 (17.07) | 0.17 |
| 30-d Unplanned readmission #, mean (SD) | 0.08 (0.27) | 0.2 (0.46) | 0.07 |
| 30-d ED visit or ED observation, n (%) | 7 (17.5) | 14 (17.07) | 0.95 |
| 30-d ED visit or ED observation, mean (SD) | 0.23 (0.58) | 0.22 (0.54) | 0.96 |
| 30-d SNF utilization days, mean (SD)[g] | - | - | |
| 30-d Home health utilization days, mean (SD)6[g] | 0.49 (1.82) | 0.61 (2) | 0.74 |
| Follow up with appointment within 14 days of discharge, n (%)[h] | 18 (54.55) | 36 (52.17) | 0.83 |

[a]Values are numbers (percentages) unless otherwise indicated. Percentages may not sum to 100 due to rounding.
[b]For home patients, includes any time they spent in the brick-and-mortar hospital.
[c]Indicates how many days a home patient was in the brick-and-mortar hospital before being transferred home.
[d]Indicates how many days a home patient was at home during their full length of stay.
[e]For 1 control patient, this data is missing.
[f]Electrocardiograms are included in imaging.
[g]For 7 control patients and 4 intervention patients, these data are missing.
[h]For 13 control patients and 7 intervention patients, these data are missing.
**Note**: see eTable 8 for comparison of use in the brick and mortar versus the use in the home in the intervention group.
Abbreviations: BAM = brick-and-mortar; ED = emergency department; LOS = length of stay; SD = standard deviation; SNF = skilled nursing facility

© 2025 Levine DM, et al. *JAMA Network Open*.

**eTable 13**. Quality, physical activity, functional status, and experience (home in less than 3 days of brick-and-mortar vs. control)[a]

| Measure | Home <3d BAM (n=40) | Control (n = 82) | p-value |
|---|---|---|---|
| *Quality and safety of care* | | | |
| Any safety event, n (%)[b, c] | 3 (7.69) | 10 (12.35) | 0.44 |
| Pain score, mean (SD)[d, e] | 1.39 (1.83) | 2.88 (2.63) | 0.001 |
| Inappropriate medication use, n (%)[f, g] | 10 (25) | 28 (34.57) | 0.30 |
| Urinary catheter use, n (%)[c] | 1 (2.56) | 3 (3.7) | 0.75 |
| Restraint use, n (%) | - | - | |
| *Activity each day* | | | |
| Percent minutes of sedentary activity, mean (SD)[h, k] | 77.96 (10.4) | 85.97 (7.17) | <0.001 |
| Percent minutes of LPA, mean (SD)[h, k] | 20.80 (9.05) | 13.92 (7.07) | 0.001 |
| Percent minutes of MVPA, mean (SD)[h, k] | 1.24 (2.2) | 0.10 (0.28) | 0.01 |
| Daily steps, mean (SD)[h] | 992.62 (1451.5) | 120.39 (206.03) | 0.003 |
| *Functional status* | | | |
| EQ-5D-5L, mean (SD)[m] | | | |
| Admission[n] | 8.36 (2.62) | 8.9 (3.16) | 0.32 |
| Discharge[o] | 7.72 (2.87) | 8.37 (3.15) | 0.28 |
| +30[p] | 9.6 (3.84) | 9.02 (4) | 0.53 |
| PHQ2, mean (SD)[q] | | | |
| Admission[c] | 1.41 (1.62) | 1.3 (1.59) | 0.71 |
| Discharge[r] | 1.09 (1.25) | 1.25 (1.27) | 0.52 |
| +30[s] | 1.5 (2.03) | 0.95 (1.44) | 0.20 |
| ADLs, mean (SD)[t] | | | |
| Admission(SE)[r] | 5.77 (0.74) | 5.6 (1.25) | 0.36 |
| Discharge(SE) | - | - | - |
| +30 | - | - | - |
| iADLs, mean (SD)[u] | | | |
| Admission[v] | 6.67 (2.06) | 6.64 (2.06) | 0.94 |
| Discharge[n] | 6.6 (2.02) | 6.39 (2.26) | 0.62 |
| +30[w] | 6.38 (2.28) | 6.57 (2.06) | 0.71 |
| Functional status change, n (%) | | | |
| ADLs worse: admission to discharge | - | - | - |
| ADLs worse: admission to 30 d after discharge | - | - | - |
| IADLs worse: admission to discharge[x] | 10 (28.57) | 19 (26.39) | 0.81 |
| IADLs worse: admission to 30 d after discharge[y] | 6 (25) | 19 (31.67) | 0.56 |
| *Patient experience* | | | |
| Picker patient experience questionnaire score, mean (SD)[z, aa] | 13.56 (2.47) | 11 (3.81) | <.001 |
| Global satisfaction score, mean (SD)[o, bb] | 9.61 (0.77) | 8.34 (1.74) | <.001 |
| Recommend care, mean (SD)[cc, dd] | 9.77 (0.65) | 8.53 (1.82) | <.001 |
| Net promoter score % (SD)[ee, dd] | 88.57 (32.28) | 45.45 (69.86) | <.001 |

Abbreviations: ADL = activity of daily living; EQ-5D-5L=EuroQol-5 Dimensions-5 Levels; iADL = instrumental ADL; IQR = interquartile range; LPA = light physical activity; MVPA = moderate to vigorous activity; PHQ2 = Patient Health Questionnaire 2; SD = standard deviation; SE = standard error

[a]Values are numbers (percentages) unless otherwise indicated.

[b]See **eTable 8** for detailed safety outcomes.

[c]For 1 control patient and 1 intervention patient, these data are missing.

[d]Range, 0 –10 where higher scores indicate greater pain.

[e]For 3 control patients, these data are missing.

[f]Using the updated Beers Criteria (29).

[g]For 1 control patient, this data is missing.

© 2025 Levine DM, et al. *JAMA Network Open*.

[h]For 29 control patients and 14 intervention patients, these data are missing. One site did not track activity data via Biofourmis.
[i]Wear time is the total time that the patient was wearing the activity device.
[j]Posture includes laying down, leaning, upright, walking and undetermined posture.
[k]Activity was categorized into sedentary, light, moderate or vigorous with sedentary being the least physical activity and vigorous being the most physical activity.
[l]Undetermined posture is any posture that could be identified as laying, leaning, upright or walking.
[m]Range, 5 – 25 where lower scores indicate less perceived health problems and higher scores indicate more perceived health problems.
[n]For 2 control patients and 1 intervention patient, these data are missing.
[o]For 6 control patients and 4 intervention patients, these data are missing.
[p]For 18 control patients and 15 intervention patients, these data are missing.
[q]Range, 0 – 6, where scores >2 indicate depression.
[r]For 7 control patients and 5 intervention patients, these data are missing.
[s]For 17 control patients and 12 intervention patients, these data are missing.
[t]Range, 0 – 6, where higher scores indicate higher patient independence.
[u]Range, 0 – 8, where higher scores indicate higher function and independence.
[v]For 5 control patients and 1 intervention patient, these data are missing.
[w]For 19 control patients and 16 intervention patients, these data are missing.
[x]For 10 control patients and 5 intervention patients, these data are missing.
[y]For 22 control patients and 16 intervention patients, these data are missing.
[z]Range 0 – 15, where higher scores indicate more problems.
[aa]For 16 control patients and 4 intervention patients, these data are missing.
[bb]Range 0 – 10, where higher scores indicate higher global satisfaction.
[cc]Range 0 – 10, where higher scores indicate more likely to recommend.
[dd]For 5 control patients and 5 intervention patients, these data are missing.
[ee]Range 0 – 100, where higher scores indicate more likely to recommend.

© 2025 Levine DM, et al. *JAMA Network Open*.

## **eAppendix 1**. Detail regarding secondary outcomes

We had poor data capture for posture (nearly half the day's hours on average could not be categorized) and instead used other prespecified and validated accelerometry measures from the patient's monitoring patch for activity including steps and activity categorization.[16–23] A patient's activity was categorized into sedentary, light physical, and moderate to vigorous such that their total categorization summed to 100% for a day. We measured cost and health care use in the 30 days after discharge using the same cost-accounting method. We tracked readmissions, distinct ED visits, primary care visits, specialist visits, home care, and skilled nursing facility use. In addition to EHR records, we supplemented with patient and caregiver reports, adding any health care received at another site.

© 2025 Levine DM, et al. *JAMA Network Open*.

**eAppendix 2.** Table 1 missing data

[a1]For 1 home patient and 1 control patient, these data are missing.
[a2]For 1 home patient, this data is missing.
[a3]For 2 home patients and 1 control patient, these data are missing.
[a4]For 3 home patients and 1 control patient, these data are missing.
[a5]For 2 home patients and 3 control patients, these data are missing.
[a6]For 2 home patients and 1 control patient, these data are missing.
[a7]For 2 home patients and 1 control patient, these data are missing.
[a8]For 2 home patients and 2 control patients, these data are missing.
[a9]For 1 home patients and 1 control patient, these data are missing.
[a10]For 3 home patients and 2 control patients, these data are missing.
[a11]For 3 home patients and 4 control patients, these data are missing.
[a12]For 1 home patient, this data is missing.

© 2025 Levine DM, et al. *JAMA Network Open*.

**eAppendix 3.** Table 4 missing data

[a1]For 1 control patients and 1 intervention patients, these data are missing.
[a2]For 3 control patients, these data are missing.
[a3]For 1 control patients and 2 intervention patients, these data are missing.
[a4]For 29 control patients and 29 intervention patients, these data are missing. One site did not track activity data via Biofourmis.
[a5]For 2 control patients and 2 intervention patients, these data are missing.
[a6]For 6 control patients and 9 intervention patients, these data are missing.
[a7]For 18 control patients and 23 intervention patients, these data are missing.
[a8]For 1 control patients and 2 intervention patients, these data are missing.
[a9]For 7 control patients and 8 intervention patients, these data are missing.
[a10]For 17 control patients and 20 intervention patients, these data are missing.
[a11]For 2 control patients and 3 intervention patients, these data are missing.
[a12]For 5 control patients and 3 intervention patients, these data are missing.
[a13]For 7 control patients and 11 intervention patients, these data are missing.
[a14]For 7 control patients and 12 intervention patients, these data are missing.
[a15]For 10 control patients and 14 intervention patients, these data are missing.
[a16]For 22 control patients and 28 intervention patients, these data are missing.
[a17]For 16 control patients and 10 intervention patients, these data are missing.
[a18]For 5 control patients and 10 intervention patients, these data are missing.

© 2025 Levine DM, et al. *JAMA Network Open*.

## eReference


1. Defining Rural Population | Official web site of the U.S. Health Resources & Services Administration. Accessed November 30, 2021. https://www.hrsa.gov/rural-health/about-us/definition/index.html2. Alberta Health Services & Alberta Health. *Official Standard Geographic Areas*. Alberta Health Service; 2018.

3. United States Census Bureau. U.S. Census Bureau QuickFacts: United States. Accessed April 22, 2024. https://www.census.gov/quickfacts/fact/table/US/POP010210

4. Profile table, Census Profile, 2021 Census of Population - Wetaskiwin, City (CY) [Census subdivision], Alberta. August 2, 2024. Accessed September 30, 2025. https://www12.statcan.gc.ca/census-recensement/2021/dp-pd/prof/details/page.cfm?Lang=E&SearchText=wetaskiwin&GENDERlist=1,2,3&STATISTIClist=1,4&DGUIDlist=2021A00054811002&HEADERlist=0

5. U.S. Bureau of Labor Statistics. Tables and Maps : U.S. Bureau of Labor Statistics. 2023. Accessed November 17, 2024. https://www.bls.gov/lau/tables.htm#cntyaa

6. Health Resources and Services Administration (HRSA). HPSA Find. Accessed April 22, 2024. https://data.hrsa.gov/tools/shortage-area/hpsa-find

7. USDA Economic Research Service. Rural-Urban Commuting Area Codes. November 4, 2024. Accessed August 31, 2022. https://www.ers.usda.gov/data-products/rural-urban-commuting-area-codes/

8. Centers for Medicare & Medicaid Services. Ambulance Fee Schedule & ZIP Code Files | CMS. November 15, 2024. Accessed April 22, 2024. https://www.cms.gov/medicare/payment/fee-schedules/ambulance

9. U.S. Department of Agriculture. USDA ERS - Frontier and Remote Area Codes. March 7, 2024. Accessed April 22, 2024. https://www.ers.usda.gov/data-products/frontier-and-remote-area-codes/

10. Health Resources and Services Administration. Rural Health Grants Eligibility Analyzer. Accessed April 22, 2024. https://data.hrsa.gov/tools/rural-health

11. Statistics Canada. Profile table, Census Profile, 2021 Census of Population - Wetaskiwin County No. 10, Municipal district (MD) [Census subdivision], Alberta. February 8, 2024. Accessed April 21, 2024. https://www12.statcan.gc.ca/census-recensement/2021/dp-pd/prof/details/page.cfm?Lang=E&SearchText=Wetaskiwin&DGUIDlist=2021A00054811001&GENDERlist=1,2,3&STATISTIClist=1,4&HEADERlist=0

12. United States Census Bureau. DP05: ACS Demographic and ... - Census Bureau Table. Accessed September 26, 2025. https://data.census.gov/table/ACSDP5Y2023.DP05?q=acs&g=050XX00US29045

13. Statistics Canada. Focus on Geography Series, 2021 Census - Wetaskiwin County No. 10 (Census subdivision). December 16, 2022. Accessed September 25, 2025. https://www12.statcan.gc.ca/census-recensement/2021/as-sa/fogs-spg/page.cfm?topic=10&lang=e&dguid=2021A00054811001

14. Exchange Rates UK. Canadian Dollar to US Dollar Spot Exchange Rates for 2022. Accessed March 3, 2025. https://www.exchangerates.org.uk/CAD-USD-spot-exchange-rates-history-2022.html

15. Exchange Rates UK. Canadian Dollar to US Dollar Spot Exchange Rates for 2023. Accessed March 3, 2025. https://www.exchangerates.org.uk/CAD-USD-spot-exchange-rates-history-2023.html

16. van Hees VT, Fang Z, Langford J, et al. Autocalibration of accelerometer data for free-living physical activity assessment using local gravity and temperature: an evaluation on four continents. *J Appl Physiol*. 2014;117(7):738-744. doi:10.1152/japplphysiol.00421.2014


© 2025 Levine DM, et al. *JAMA Network Open*.

17. van Hees VT, Sabia S, Anderson KN, et al. A Novel, Open Access Method to Assess Sleep Duration Using a Wrist-Worn Accelerometer. *PLoS ONE*. 2015;10(11):e0142533. doi:10.1371/journal.pone.0142533

18. van Hees VT, Sabia S, Jones SE, et al. Estimating sleep parameters using an accelerometer without sleep diary. *Sci Rep*. 2018;8(1):12975. doi:10.1038/s41598-018-31266-z

19. van Hees VT, Migueles JH, Sabia S, et al. CRAN: Package GGIR. February 20, 2025. Accessed January 8, 2025. https://cran.r-project.org/web/packages/GGIR/index.html

20. GitHub - walkabillylab/activityCounts: Creates ActiLife counts based on the raw acceleration data. Accessed January 7, 2025. https://github.com/walkabillylab/activityCounts

21. GitHub - dipetkov/actigraph.sleepr: Detect periods of sleep and non-wear from ActiGraph data. Accessed January 7, 2025. https://github.com/dipetkov/actigraph.sleepr

22. Choi L, Beck C, Liu Z, Moore R, Matthews CE, Buchowski MS. CRAN: Package PhysicalActivity. January 22, 2021. Accessed January 8, 2025. https://cran.r-project.org/web/packages/PhysicalActivity/index.html

23. Migueles JH, Rowlands AV, Huber F, Sabia S, van Hees VT. GGIR: A Research Community–Driven Open Source R Package for Generating Physical Activity and Sleep Outcomes From Multi-Day Raw Accelerometer Data. *Journal for the Measurement of Physical Behaviour*. 2019;2(3):188-196. doi:10.1123/jmpb.2018-0063

© 2025 Levine DM, et al. *JAMA Network Open*.
