# Supplementary material for: Hospital-Level Care at Home for Adults Living in Rural Settings: A Randomized Clinical Trial
Source: JAMA Netw Open. 2025 Dec 1;8(12):e2545712. doi: 10.1001/jamanetworkopen.2025.45712 (PMC12670196; doi:10.1001/jamanetworkopen.2025.45712)
Supplement: Supplement 3. — Data Sharing Statement [file jamanetwopen-e2545712-s003.pdf]

Data Sharing Statement

Levine. Hospital-Level Care at Home for Adults Living in Rural Settings. *JAMA Netw Open*. Published December 01, 2025. doi:10.1001/jamanetworkopen.2025.45712

Data
Additional Information: Title: Rural Hospital-Level Care at Home for Acutely Ill Adults URL: https://clinicaltrials.gov/study/NCT05256303 Trial Registration Number: NCT05256303
Data available: No
